# Preferential association of a functional variant in complement receptor 2 with antibodies to double-stranded DNA

**DOI:** 10.1136/annrheumdis-2014-205584

**Published:** 2014-09-01

**Authors:** Jian Zhao, Brendan M Giles, Rhonda L Taylor, Gabriel A Yette, Kara M Lough, Han Leng Ng, Lawrence J Abraham, Hui Wu, Jennifer A Kelly, Stuart B Glenn, Adam J Adler, Adrienne H Williams, Mary E Comeau, Julie T Ziegler, Miranda Marion, Marta E Alarcón-Riquelme, for the BIOLUPUS and GENLES Networks, Graciela S Alarcón, Juan-Manuel Anaya, Sang-Cheol Bae, Dam Kim, Hye-Soon Lee, Lindsey A Criswell, Barry I Freedman, Gary S Gilkeson, Joel M Guthridge, Chaim O Jacob, Judith A James, Diane L Kamen, Joan T Merrill, Kathy Moser Sivils, Timothy B Niewold, Michelle A Petri, Rosalind Ramsey-Goldman, John D Reveille, R Hal Scofield, Anne M Stevens, Luis M Vilá, Timothy J Vyse, Kenneth M Kaufman, John B Harley, Carl D Langefeld, Patrick M Gaffney, Elizabeth E Brown, Jeffrey C Edberg, Robert P Kimberly, Daniela Ulgiati, Betty P Tsao, Susan A Boackle

**Affiliations:** 1 Division of Rheumatology, Department of Medicine, University of California at Los Angeles, Los Angeles, California, USA; 2 Division of Rheumatology, University of Colorado School of Medicine, Aurora, Colorado, USA; 3 School of Pathology and Laboratory Medicine, Centre for Genetic Origins of Health and Disease, The University of Western Australia, Crawley, Western Australia, Australia; 4 Arthritis and Clinical Immunology Research Program, Oklahoma Medical Research Foundation, Oklahoma City, Oklahoma, USA; 5 Department of Biostatistical Sciences and Center for Public Health Genomics, Wake Forest School of Medicine, Winston-Salem, North Carolina, USA; 6 Pfizer-Universidad de Granada-Junta de Andalucía Center for Genomics and Oncological Research, Granada, Spain; 7 Department of Medicine, University of Alabama at Birmingham, Birmingham, Alabama, USA; 8 Center for Autoimmune Diseases Research (CREA), Universidad del Rosario, Bogotá, Colombia; 9 Department of Rheumatology, Hanyang University Hospital for Rheumatic Diseases, Seoul, South Korea; 10 Rosalind Russell/Ephraim P. Engleman Rheumatology Research Center, University of California San Francisco, San Francisco, California, USA; 11 Department of Internal Medicine, Wake Forest School of Medicine, Winston-Salem, North Carolina, USA; 12 Division of Rheumatology, Medical University of South Carolina, Charleston, South Carolina, USA; 13 Department of Medicine, University of Southern California, Los Angeles, California, USA; 14 Department of Pathology, University of Oklahoma Health Sciences Center, Oklahoma City, Oklahoma, USA; 15 Department of Medicine, University of Oklahoma Health Sciences Center, Oklahoma City, Oklahoma, USA; 16 Department of Clinical Pharmacology, Oklahoma Medical Research Foundation, Oklahoma City, Oklahoma, USA; 17 Division of Rheumatology and Department of Immunology, Mayo Clinic, Rochester, Minnesota, USA; 18 Department of Medicine, Johns Hopkins University School of Medicine, Baltimore, Maryland, USA; 19 Division of Rheumatology, Northwestern University Feinberg School of Medicine, Chicago, Illinois, USA; 20 Department of Rheumatology and Clinical Immunogenetics, University of Texas Health Science Center at Houston, Houston, Texas, USA; 21 US Department of Veterans Affairs Medical Center, Oklahoma City, Oklahoma, USA; 22 Division of Rheumatology, Department of Pediatrics, University of Washington, Seattle, Washington, USA; 23 Center for Immunity and Immunotherapies, Seattle Children's Research Institute, Seattle, Washington, USA; 24 Division of Rheumatology, Department of Medicine, University of Puerto Rico Medical Sciences Campus, San Juan, Puerto Rico; 25 Division of Genetics and Molecular Medicine and Immunology, King's College London, London, UK; 26 Cincinnati Children's Hospital Medical Center, Cincinnati, Ohio, USA; 27 US Department of Veterans Affairs Medical Center, Cincinnati, Ohio, USA; 28 Department of Epidemiology, University of Alabama at Birmingham, Birmingham, Alabama, USA; 29 Denver Veterans Affairs Medical Center, Denver, Colorado, USA

**Keywords:** Systemic Lupus Erythematosus, Autoantibodies, Gene Polymorphism, B cells

## Abstract

**Objectives:**

Systemic lupus erythematosus (SLE; OMIM 152700) is characterised by the production of antibodies to nuclear antigens. We previously identified variants in complement receptor 2 (*CR2/CD21*) that were associated with decreased risk of SLE. This study aimed to identify the causal variant for this association.

**Methods:**

Genotyped and imputed genetic variants spanning *CR2* were assessed for association with SLE in 15 750 case-control subjects from four ancestral groups. Allele-specific functional effects of associated variants were determined using quantitative real-time PCR, quantitative flow cytometry, electrophoretic mobility shift assay (EMSA) and chromatin immunoprecipitation (ChIP)-PCR.

**Results:**

The strongest association signal was detected at rs1876453 in intron 1 of *CR2* (p_meta_=4.2×10^−4^, OR 0.85), specifically when subjects were stratified based on the presence of dsDNA autoantibodies (case-control p_meta_=7.6×10^−7^, OR 0.71; case-only p_meta_=1.9×10^−4^, OR 0.75). Although allele-specific effects on B cell *CR2* mRNA or protein levels were not identified, levels of complement receptor 1 (*CR1/CD35)* mRNA and protein were significantly higher on B cells of subjects harbouring the minor allele (p=0.0248 and p=0.0006, respectively). The minor allele altered the formation of several DNA protein complexes by EMSA, including one containing CCCTC-binding factor (CTCF), an effect that was confirmed by ChIP-PCR.

**Conclusions:**

These data suggest that rs1876453 in *CR2* has long-range effects on gene regulation that decrease susceptibility to lupus. Since the minor allele at rs1876453 is preferentially associated with reduced risk of the highly specific dsDNA autoantibodies that are present in preclinical, active and severe lupus, understanding its mechanisms will have important therapeutic implications.

## Introduction

Systemic lupus erythematosus (SLE (OMIM 152700)) is a heterogeneous autoimmune disease with a strong genetic component modified by environmental exposures. The complement system has been linked to its pathogenesis since low serum complement levels were first demonstrated in patients with active disease.^1^ ^2^ Although complement activation resulting in tissue damage is an important feature of lupus, deficiencies of early classical complement pathway components are paradoxically strongly associated with lupus susceptibility. Deficiencies or altered function of complement receptors have also been associated with lupus in humans and murine models of disease.^3–5^ These effects have been attributed to alterations in antigen clearance, antigen processing, tolerance induction and cell activation, but the exact mechanisms remain poorly understood.

SLE is characterised by the production of class-switched autoantibodies against nuclear antigens that have undergone affinity maturation, suggesting their generation in germinal centre reactions. Complement receptor 2 (*CR2/CD21*) is primarily expressed on mature B cells and follicular dendritic cells, two major components of germinal centres. We first showed *Cr2*, which encodes both CR2 and complement receptor 1 (CR1/CD35) in the mouse, to be a candidate gene for lupus susceptibility in the NZM2410 model of lupus based on structural and functional alterations in its gene products.^6^ We subsequently demonstrated strong association of a common three single-nucleotide polymorphism (SNP) *CR2* haplotype (rs3813946 in the 5′UTR, rs1048971 and rs17615 in exon 10) with increased risk of lupus susceptibility (p=1.0×10^−5^) in Caucasian and Chinese lupus simplex families with a 1.54-fold increased risk for disease development.^7^ We confirmed this in a case-control analysis of an independent European-derived population (p=2.3×10^−2^, OR 1.1 (95% CI 1.02 to 1.2))^8^ and also showed that a haplotype formed by the minor alleles of three SNPs in exons 10 and 11 (rs1048971, rs17615, rs4308977) was associated with decreased risk of lupus (p=1.6×10^−2^, OR 0.90 (95% CI 0.82 to 0.98)).^8^

In this study, we fine-mapped the region spanning *CR2* in 15 750 subjects from four ancestral groups to identify potential causal variant(s) for these associations with lupus. Additionally, we explored the association of *CR2* polymorphisms with clinical manifestations of lupus in order to generate new hypotheses regarding how CR2 contributes to disease development.

## Methods

### Subjects

DNA from individuals recruited from multiple sites was processed at the Oklahoma Medical Research Foundation (OMRF; Large Lupus Association Study 2) with institutional review board approval. All patients with SLE met the 1997 American College of Rheumatology revised classification criteria.^9^ Clinical data were collected by chart review or testing in the OMRF Clinical Immunology laboratory. Samples for functional analyses were from healthy non-smoking 18-year-old to 60-year-old adults without family history of autoimmune disease at the University of Colorado School of Medicine.

### Genotyping

Genotyping was performed on the OMRF Illumina iSelect platform.^10^ ^11^ Subjects with missing genotype rate >10%, shared identical by descent >0.4 or gender mismatch were removed. Global ancestry was estimated based on the genotype of ancestry informative markers (AIMs), using principal components analysis^12^ and ADMIXMAP^13^ as described^14^ and genetic outliers removed. Final clean data were from European Americans (EA), African Americans (AA; 7.5% Gullahs), Asians (AS; 74.6% Koreans, 16.1% Chinese, 9.3% Japanese and Singaporeans) and Hispanics (HS) enriched for Amerindian–European admixture. 2001 EA cases and 2153 EA controls were previously analysed.^8^ Subjects for functional studies were genotyped using a Taqman SNP Genotyping Assay.

### Imputation

SNP and insertion-deletion (INDEL) genotypes of 379 Europeans, 246 Africans, 286 Asians and 181 Americans from the 1000 Genomes Project (V.3, Phase 1 integrated data, March 2012 release) were references in imputation for EA, AA, AS and HS subjects, respectively. Imputation was performed using IMPUTE 2.1.2^15^; genotypes with information scores >0.9 and minor allele frequency (MAF)>0.01 were further analysed.

### Association tests

SNPs and INDELs showing biased Hardy–Weinberg equilibrium (p<0.01 in controls, p<0.0001 in cases), missing genotype rate >5% or different missing genotype rates between cases and controls (rate >2% and p<0.05) were excluded. Variants were assessed for association with SLE under a logistic regression model, and haplotypic and haplotype-based conditional association tests were performed, adjusting for gender and the first three principal components estimated using AIMs. For transancestral meta-analysis, a fixed effect model was applied if Cochran's Q statistic showed no evidence of genetic heterogeneity among ORs (p>0.05); otherwise, a random effect model was used. Analyses were performed using PLINK V.1.07.^16^

### Sample preparation

Peripheral blood mononuclear cells (PBMC) were isolated over Ficoll–Paque (Sigma–Aldrich). DNA was purified using the QIAamp DNA Mini Kit (QIAgen). B cells were purified using the Easy Sep Human B Cell Enrichment Kit (StemCell Technologies). RNA was processed using the RNeasy Plus Mini Kit (QIAgen). Quality check and quantification of RNA was performed using the Agilent 2100 Bioanalyzer. RNA and DNA were stored at −70°C. Epstein–Barr virus (EBV)-transformed B cell lines were generated by incubating PBMCs with supernatant from cell line GM7404A and cyclosporine A.

### qPCR and quantitative flow cytometry

qPCR of primary B cell transcripts was performed using cDNA transcribed using random primers and MultiScribe reverse transcriptase (Applied Biosystems), customised *CR2* primers (5′-CGAGAAGTATATTCTGTTGATCCATACAA-3′, 5′-CTAATCAATATTCCGCTGAATTCCA-3′) and probe (6FAM-AACTGGTGTGTGCCTCA-MGBNFQ), Taqman assays for *CR1* (4331182) and β*-actin* (4352935E), and the Applied Biosystems 7500 Real-Time PCR System. Relative expression levels of *CR2* and *CR1*, normalised to β*-actin*, were calculated using the comparative C_T_ method.^17^

Quantitative flow cytometry was performed using Quantum Simply Cellular microbeads (Bangs Laboratories). Purified B cells were incubated with ethidium monoazide to exclude dead cells and mouse IgG to block Fc receptors and stained with biotinylated anti-human CD19 (clone HD37) followed by AF405-labelled streptavidin (Molecular Devices) and either AF488-labelled anti-human CR1 (clone 6B1.H12) or AF488-labelled anti-human CR2 (clone THB5, Harlan). Data acquired on fixed cells within 24 h using the LSR II and CellQuest software (BD Biosciences) were analysed with FlowJo (Tree Star).

### Electrophoretic mobility shift assay

39-mer oligonucleotides **(**5′–GGGCCAAAAGCGAGACGGT[G/A]GGGGCAGTGCTCGACG–3′, 5′–CGTCGAGCACTGCCCC[C/T]ACCGTCTCGCTTTTGGCCC–3′) were synthesised, HPLC-purified and biotin-labelled; 25 fmol labelled oligonucleotides were incubated with 2–4 μg nuclear extracts (NE) or 50 mM potassium chloride (for unbound controls) in 4% Ficoll, 20 mM N-2-hydroxyethylpiperazine-N-2-ethane sulfonic acid (HEPES), 1 mM EDTA, 0.5 mM dithiothreitol and 1 µg poly dI:dC for 30 min on ice. For competition and blocking experiments, NE were pre-incubated with unlabelled oligonucleotide or anti-CCCTC-binding factor (CTCF) (sc-15914; Santa Cruz Biotechnology) for 10 or 30 min on ice. Reactions were electrophoresed on 6% DNA retardation gels (Life Technologies), transferred and cross-linked using ultraviolet light to nylon membranes and visualised using the Chemiluminescent Nucleic Acid Detection Module (Thermo Fisher Scientific).

### Chromatin immunoprecipitation-PCR

Chromatin immunoprecipitation (ChIP) was performed using the Upstate Biotechnology ChIP assay kit with minor modifications.^18^ 1.1×10^8^ cells were incubated with 1% formaldehyde for 15 min and 0.125 M glycine added for 5 min. Washed cells were incubated in NP-40 lysis buffer and nuclei pelleted, resuspended in sodium dodecyl sulfate lysis buffer and sonicated five times on ice. Precleared soluble chromatin was incubated with anti-CTCF, an isotype control, or no antibody followed by Protein G agarose/salmon sperm DNA. qPCR was performed on immune complex-associated DNA using primers spanning rs1876453 (5′–GGAAAGTTTCTGTGCCGCGA–3′, 5′-GACAATCAGGACCAGGCGGT–3′), SYBR Green-based detection, and the Illumina Eco Real-Time PCR System. A standard curve was constructed using a chromatin input control.

qPCR of EBV-transformed cell transcripts was performed using cDNA transcribed using the Superscript VILO cDNA synthesis kit (Life Technologies), primers specific for β*-actin* (5′-GATGACCCAGATCATGTTTGAG-3′, 5′-GACTCCATGCCCAGGAAGGAA-3′), *CTCF* (5′-CAACCAGCCCAAACAGAACCAG-3′, 5′-TCCTCTTCCTCTCCCTCTGC-3′), *CR2* (5′-TGCCTGTAAAACCAACTTCTC-3′, 5′-AGCAAGTAACCAGATTCACAG-3′) and *CR1* (5′-TGCTAAGGACAGGTGCAGAC-3′, 5′-GGCAGACGAGGAACCAATGA-3′), SYBR Green-based detection, and the Eco Real-Time PCR System (Illumina). Transcript abundance was determined by comparison with a standard curve and normalised to β*-actin*.

### Statistical analysis

A two-tailed unpaired Student t test detected differences between groups. Spearman rank correlation determined correlation within a group. A p value of <0.05 was considered significant. Statistics and graphs were generated using GraphPad Prism software.

## Results

### Association of rs1876453 with decreased risk of lupus

To localise causal variants of the *CR2* gene, we genotyped 56 SNPs in *CR2* and *CR1* and 347 AIMs in 15 750 unrelated case-control subjects from four ancestral groups: EA (3872 cases vs 3449 controls), AA (1676 vs 1929), AS (1265 vs 1260) and HS (1492 vs 807). Genotypes for additional variants (SNPs and INDELs) were imputed using reference data from the 1000 Genomes Project. After applying quality control measures, 138 EA, 167 AA, 102 AS and 133 HS genetic variants deeply covering a 57.6 kB region 5′ upstream of *CR2* to intron 1 of *CR1* were assessed for association with SLE (see online supplementary figure S1 and table S1). Only rs1876453, in intron 1 of *CR2*, was consistently associated with SLE in EA (MAF 0.086 in cases vs 0.099 in controls, p=0.045, OR 0.89 (95% CI 0.79 to 0.99)), AA (0.075 vs 0.086, p=0.045, OR 0.83 (95% CI 0.70 to 0.99)) and HS (0.032 vs 0.051, p=3.5×10^−3^, OR 0.62 (95% CI 0.46 to 0.86)). rs1876453 was not associated with SLE in AS given the low MAF in this group (0.0004 vs 0.0008, p=0.68, OR 0.60 (95% CI 0.05 to 6.74)). In transancestral meta-analysis of 75 genetic variants assessed in EA, AA, AS and HS (see online supplementary figure S1 and table S1), the strongest association signal was detected at rs1876453 (p=4.2×10^−4^, OR 0.85) and only this signal reached the Bonferroni-corrected significance level (p<6.7×10^−4^).

### Increased signal associated with rs1876453 after subphenotype stratification

We next assessed the association of rs1876453 with 14 lupus subphenotypes. In case controls, the minor allele was consistently associated with decreased risk of serositis, renal disorder, haematological disorder and dsDNA autoantibodies in EA, AA and HS, of which the strongest association was detected with dsDNA autoantibodies (p_EA_=6.6×10^−4^, OR 0.73 (95% CI 0.61 to 0.88); p_AA_=9.7×10^−3^, OR 0.72 (95% CI 0.56 to 0.92); p_HS_=7.1×10^−3^, OR 0.58 (95% CI 0.39 to 0.86); p_meta_=7.6×10^−7^, OR 0.71) (table 1). In cases only, the minor allele of rs1876453 was associated with decreased risk of dsDNA autoantibodies (p_meta_=1.9×10^−4^, OR 0.75) and renal disorder (p_meta_=1.3×10^−2^, OR 0.83) (table 1), but only the association with dsDNA autoantibodies remained significant after correction for multiple comparisons.

**Table 1:** Association of rs1876453 with 14 SLE subphenotypes

Subphenotypes	N			MAF			Pos vs Control		Pos vs Neg	
	Group	Pos	Neg	Pos	Neg	Control	p Value	OR (95% CI)	p Value	OR (95% CI)
Malar rash	EA	2108	1171	0.088	0.087	0.099	0.11	0.89 (0.78 to 1.03)	0.92	1.01 (0.85 to 1.21)
	AA	685	714	0.08	0.066	0.086	0.24	0.87 (0.69 to 1.10)	0.15	1.24 (0.92 to 1.65)
	HS	836	572	0.03	0.032	0.051	***3***.***0*×*10^−3^***	0.57 (0.39 to 0.83)	0.62	0.89 (0.57 to 1.40)
	Meta						***5***.***7*×*10^−3^***	0.85	0.52	1.05
Discoid rash	EA	573	2486	0.088	0.088	0.099	0.38	0.91 (0.73 to 1.13)	0.98	1.00 (0.80 to 1.25)
	AA	485	915	0.088	0.065	0.086	0.81	0.97 (0.75 to 1.25)	***0***.***029***	1.39 (1.04 to 1.86)
	HS	180	1227	0.026	0.031	0.051	***0***.***032***	0.46 (0.22 to 0.93)	0.43	0.75 (0.37 to 1.53)
	Meta						0.2	0.9	0.26	1.1
Photosensitivity	EA	2345	1142	0.091	0.08	0.099	0.29	0.93 (0.82 to 1.06)	0.16	1.14 (0.95 to 1.36)
	AA	685	712	0.078	0.069	0.086	0.14	0.84 (0.66 to 1.06)	0.39	1.14 (0.85 to 1.52)
	HS	840	564	0.037	0.022	0.051	***0***.***047***	0.70 (0.49 to 0.99)	***0***.***05***	1.64 (1.00 to 2.67)
	Meta						***0***.***029***	0.89	***0***.***031***	1.17
Oral ulcers	EA	1574	1642	0.09	0.09	0.099	0.22	0.91 (0.78 to 1.06)	0.9	0.99 (0.83 to 1.17)
	AA	495	903	0.059	0.081	0.086	***1***.***7*×*10^−3^***	0.63 (0.47 to 0.84)	0.06	0.74 (0.54 to 1.01)
	HS	562	844	0.036	0.027	0.051	0.094	0.71 (0.48 to 1.06)	0.2	1.33 (0.85 to 2.08)
	Meta						***3***.***5*×*10^−3^***	0.83	0.59	0.96
Arthritis	EA	2987	619	0.088	0.091	0.099	0.075	0.89 (0.79 to 1.01)	0.76	0.97 (0.78 to 1.20)
	AA	1166	232	0.076	0.059	0.086	***0***.***048***	0.82 (0.67 to 1.01)	0.19	1.33 (0.87 to 2.02)
	HS	1007	402	0.031	0.031	0.051	***3***.***7*×*10^−3^***	0.60 (0.42 to 0.85)	0.96	1.01 (0.62 to 1.66)
	Meta						***9***.***9*×*10^−4^***	0.84	0.76	1.03
Serositis	EA	1341	2060	0.084	0.092	0.099	***0***.***042***	0.85 (0.72 to 0.99)	0.26	0.91 (0.76 to 1.08)
	AA	653	742	0.07	0.076	0.086	***0***.***025***	0.75 (0.59 to 0.96)	0.55	0.92 (0.69 to 1.22)
	HS	384	877	0.029	0.03	0.051	***0***.***022***	0.56 (0.34 to 0.92)	0.94	0.98 (0.58 to 1.64)
	Meta						***6***.***0*×*10^−4^***	0.8	0.21	0.91
Renal disorder	EA	1102	2143	0.079	0.098	0.099	***0***.***016***	0.80 (0.67 to 0.96)	***0***.***018***	0.80 (0.67 to 0.96)
	AA	697	701	0.065	0.081	0.086	***6***.***1*×*10^−3^***	0.71 (0.55 to 0.91)	0.092	0.78 (0.58 to 1.04)
	HS	644	746	0.032	0.028	0.051	***0***.***029***	0.64 (0.43 to 0.96)	0.41	1.21 (0.77 to 1.91)
	Meta						***4***.***4*×*10^−5^***	0.75	***0***.***013***	0.83
Neurological disorder	EA	575	2524	0.099	0.092	0.099	0.89	1.02 (0.82 to 1.26)	0.51	1.07 (0.87 to 1.33)
	AA	372	1025	0.088	0.068	0.086	0.75	0.95 (0.72 to 1.27)	0.069	1.34 (0.98 to 1.82)
	HS	194	1213	0.024	0.032	0.051	***0***.***036***	0.47 (0.23 to 0.95)	0.42	0.75 (0.37 to 1.52)
	Meta						0.58	0.95	0.18	1.12
Haematological disorder	EA	2173	1081	0.085	0.089	0.099	***0***.***038***	0.87 (0.76 to 0.99)	0.67	0.96 (0.80 to 1.15)
	AA	1011	384	0.07	0.079	0.086	***0***.***012***	0.76 (0.62 to 0.94)	0.45	0.89 (0.65 to 1.22)
	HS	811	459	0.031	0.028	0.051	***9***.***6*×*10^−3^***	0.61 (0.42 to 0.89)	0.79	1.07 (0.65 to 1.77)
	Meta						***1***.***9*×*10^−4^***	0.81	0.53	0.95
Anti-dsDNA	EA	1179	1500	0.071	0.104	0.099	***6***.***6*×*10^−4^***	0.73 (0.61 to 0.88)	***1***.***2×10^−4^***	0.68 (0.56 to 0.83)
	AA	675	746	0.067	0.083	0.086	***9***.***7*×*10^−3^***	0.72 (0.56 to 0.92)	0.097	0.79 (0.59 to 1.04)
	HS	726	564	0.03	0.028	0.051	***7***.***1*×*10^−3^***	0.58 (0.39 to 0.86)	0.61	1.13 (0.70 to 1.84)
	Meta						***7***.***6*×*10^−7^***	0.71	***1***.***9×10^−4^***	0.75
Anti-Sm	EA	225	2064	0.109	0.093	0.099	0.37	1.15 (0.85 to 1.56)	0.24	1.20 (0.88 to 1.64)
	AA	430	755	0.087	0.076	0.086	0.78	0.96 (0.74 to 1.26)	0.41	1.14 (0.84 to 1.54)
	HS	294	905	0.03	0.029	0.051	0.069	0.60 (0.35 to 1.04)	0.71	1.11 (0.63 to 1.98)
	Meta						0.78	0.97	0.15	1.16
Anti-RNP	EA	255	1382	0.104	0.095	0.099	0.46	1.12 (0.83 to 1.51)	0.49	1.12 (0.82 to 1.53)
	AA	378	253	0.078	0.077	0.086	0.34	0.87 (0.65 to 1.16)	0.91	1.03 (0.67 to 1.57)
	HS	171	443	0.024	0.04	0.051	***0***.***033***	0.44 (0.21 to 0.94)	0.16	0.57 (0.26 to 1.26)
	Meta						0.45	0.93	0.86	1.02
Anti-SSA/Ro	EA	450	1331	0.086	0.093	0.099	0.28	0.87 (0.68 to 1.12)	0.51	0.92 (0.70 to 1.19)
	AA	292	542	0.062	0.08	0.086	***0***.***023***	0.66 (0.46 to 0.94)	0.21	0.77 (0.51 to 1.15)
	HS	238	491	0.028	0.039	0.051	***0***.***027***	0.51 (0.28 to 0.93)	0.28	0.70 (0.36 to 1.34)
	Meta						***5***.***4*×*10^−3^***	0.76	0.13	0.85
Anti-SSB/La	EA	148	1506	0.091	0.095	0.099	0.76	0.94 (0.62 to 1.41)	0.87	0.97 (0.64 to 1.46)
	AA	81	692	0.037	0.08	0.086	***0***.***025***	0.39 (0.17 to 0.89)	0.057	0.44 (0.19 to 1.03)
	HS	71	558	0.042	0.033	0.051	0.63	0.81 (0.34 to 1.92)	0.53	1.33 (0.54 to 3.30)
	Meta						0.18	0.79	0.5	0.89

N, number in sample; MAF, minor allele frequency; Pos, subphenotype positive; Neg, subphenotype negative; p value, p value and OR were calculated using a logistic regression model adjusted for gender and global ancestry; EA, European–American; AA, African–American; HS, Hispanic; Meta, Meta-analysis; SLE, systemic lupus erythematosus. p Values ≤0.05 are marked with bold italics; significant p values after Bonferroni correction were ≤3.6×10^−3^.

### rs1876453 tags the protective EA haplotype

**Figure 1:**
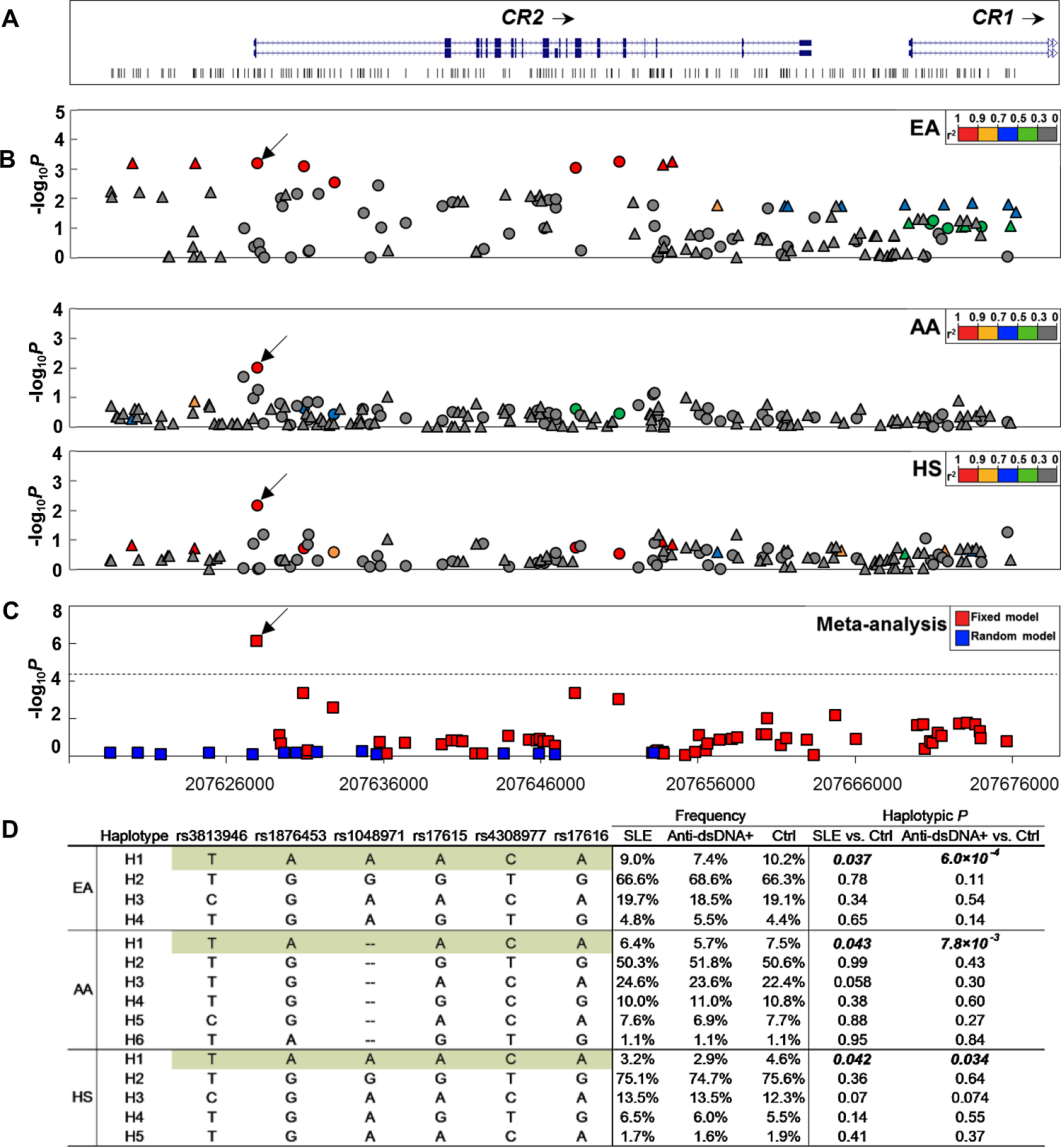
Association of single-nucleotide polymorphisms (SNPs) in the *CR2* region with dsDNA autoantibodies. (A) The genomic structure of the *CR2* region and positions of genetic variants are indicated. (B) The allelic p value (-log_10_p value) of each genetic variant with dsDNA autoantibodies is plotted against its position as a circle (genotyped) or a triangle (imputed) for European American (EA), African American (AA) and Hispanic (HS), respectively. Genetic variants are highlighted using different colours according to their strength of linkage disequilibrium (LD) (r^2^) with rs1876453. An arrow is used to indicate the position of rs1876453. (C) Transancestral meta-analysis p value generated using fixed and random model are highlighted as red and blue, respectively. The dashed line represents the significance level after Bonferroni correction. (D) Frequencies, ORs and p values of haplotypes formed by lupus-associated *CR2* SNPs in various ancestral groups. Haplotype H1 corresponds to the previously reported systemic lupus erythematosus (SLE)-associated haplotype and is highlighted in green.

No other SNP in this region exhibited stronger association with dsDNA autoantibodies in EA, AA and HS than rs1876453 (figure 1A–C, see online supplementary table S2). When conditioning on rs1876453, the association signals at all other SNPs were eliminated (see online supplementary table S2). The H1 haplotype, constructed using rs1876453 and previously associated or tightly linked *CR2* SNPs, was equivalent to the protective EA SLE haplotype^8^ and was perfectly tagged in EA by the minor allele of rs1876453 (figure 1D). In all three ancestral groups, it was associated with decreased risk of SLE (p_EA_=0.037, p_AA_=0.043 and p_HS_=0.042) and dsDNA autoantibodies (p_EA_=6.0×10^−4^, p_AA_=7.8×10^−3^ and p_HS_=0.034).

### The minor allele at rs1876453 is associated with altered expression of B cell CR1

**Figure 2:**
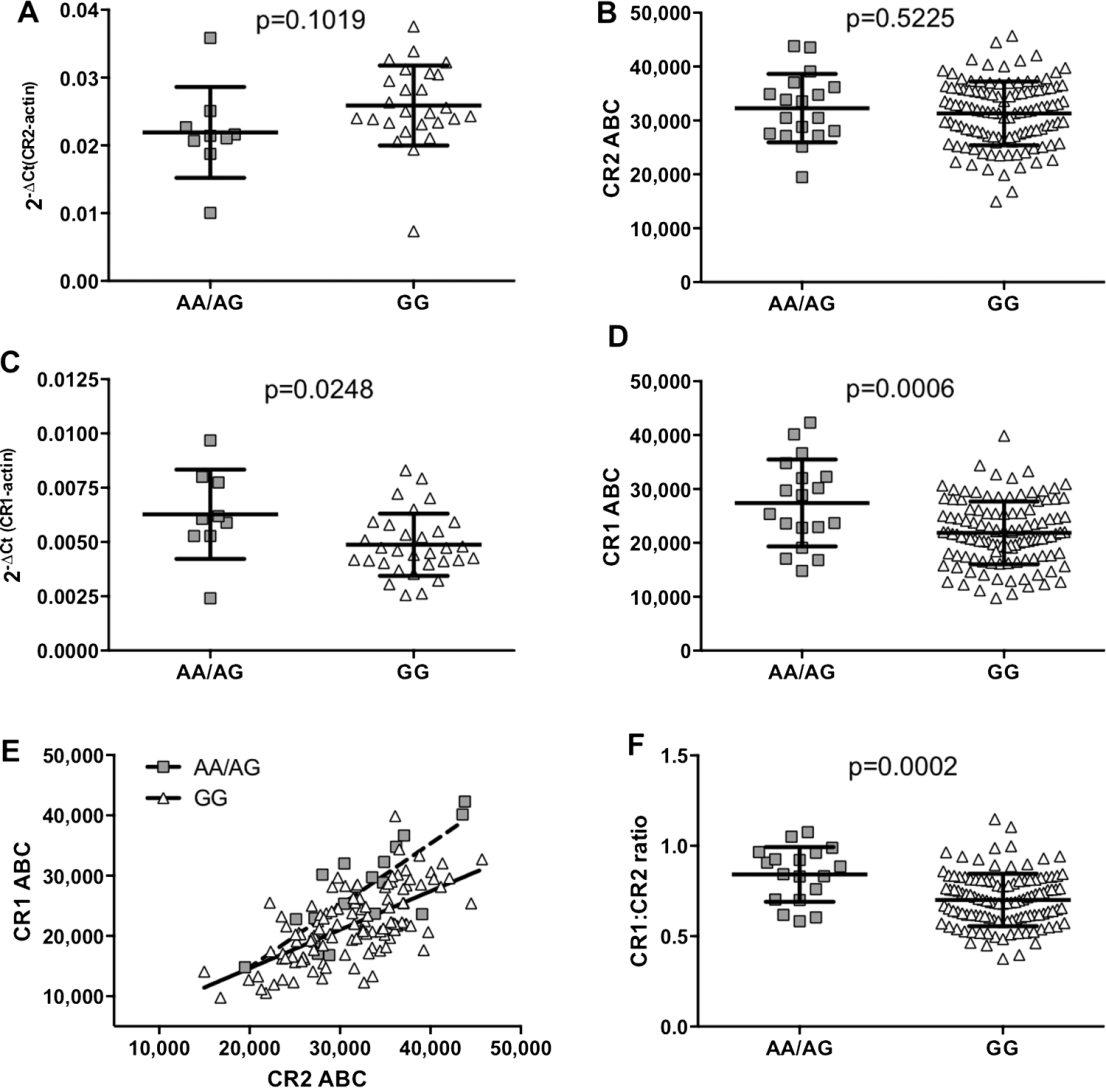
Allele-specific effects of rs1876453 on *CR1* and *CR2* expression. Relative amounts of *CR2* (A) or *CR1* (C) RNA transcripts in primary B cells from 35 or 40 healthy donors respectively were measured by qPCR using the comparative Ct method.^17^ Levels of surface CR2 (B) and CR1 (D) on primary B cells from 131 healthy donors were determined by quantitative flow cytometry (ABC, antibody binding capacity). Each point represents a unique subject, and the line and error bars represent the mean SD for each group; p values were determined using a two-tailed Student t test, and a p value of <0.05 was considered significant. (E) Levels of surface CR1 and CR2 were plotted and subjected to correlation analysis and linear regression. Each point represents a unique subject (minor allele, squares; major allele, triangles) and the lines represent the line of best fit for each allele (minor allele, dashed line; major allele, solid line). (F) Levels of surface CR1 and CR2 were also used to calculate the ratio of CR1 to CR2. Each point represents a unique subject, and the line and error bars represent the mean±SD for each group; p values were determined using a two-tailed Student t test and a p value of <0.05 was considered significant.

We did not detect allele-specific differences in *CR2* mRNA or protein levels in primary B cells from healthy controls (figure 2A, B). However, *CR1* mRNA and protein levels were significantly higher in subjects with the minor A allele (figure 2C, D). Although B cell CR1 and CR2 levels were positively correlated in both groups (major allele Spearman r=0.6342, p<0.0001; minor allele Spearman r=0.7998, p<0.0001, figure 2E), the ratio of CR1 to CR2 on B cells was higher in subjects with the minor allele (p=0.0002; figure 2F). Allele-specific differences in CR1 expression were not detected on other peripheral blood cells (see online supplementary figure S2). These data suggest that rs1876453 has long-range effects on the regulation of expression of *CR1*, which lies directly 3′ of *CR2*, that are either B cell-specific or dependent on coexpression of CR2.

### The minor allele at rs1876453 alters transcription factor binding

**Figure 3:**
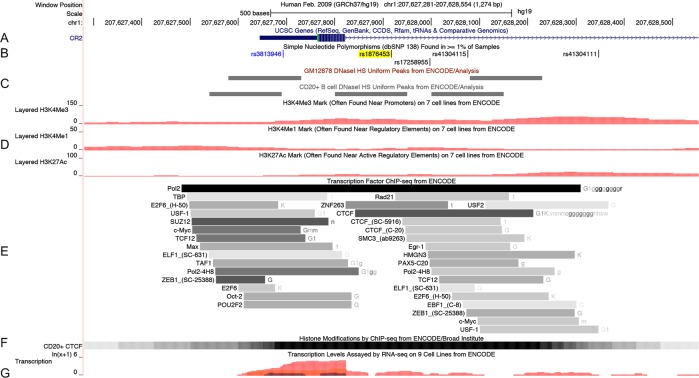
The ENCyclopedia Of DNA Elements (ENCODE) Project data surrounding rs1876453. (A) The first exon and 5′ end of the first intron of the *CR2* gene. The 5’ untranslated region (5′ UTR) is shown before the methionine start codon, in green. These data are derived from the University of California Santa Cruz (UCSC) Genes Track. (B) The location of rs1876453 (highlighted in yellow) and previously reported systemic lupus erythematosus-associated rs3813946 (in blue font). These data are derived from the Common Single-Nucleotide Polymorphisms (SNP) (138) Track (ft.ncbi.nih.gov/snp).^39^ (C) DNaseI hypersensitive sites in the GM12878 Epstein–Barr virus (EBV)-transformed B cell line and in primary CD20+ B cells derived by DNase-seq. These data are derived from the UCSC Uniform DNaseI HS Track. Signal values are shown as grayscale-coloured items where higher signal values correspond to darker-coloured blocks. Primary B cells contain an additional hypersensitivity site that overlies rs1876453. (D) Histone marks surrounding rs1876453, as determined by chromatin immunoprecipitation (ChIP)-seq. These data were derived from the Layered H3K4Me3, H3K4Me1 and H3K27Ac Tracks. The H3K4Me3 histone mark is associated with poised or active promoters, the H3K4me1 histone mark is associated with enhancers and with DNA regions downstream of transcription sites and the H3K27Ac histone mark may enhance transcription by blocking the spread of the repressive histone mark H3K27Me3. Data shown are for the GM12878 EBV-transformed B cell line. (E) Transcription factor binding sites determined by ChIP-seq are shown as grey boxes that encompass the peaks of transcription factor occupancy, with the darkness of the box proportional to the maximal signal strength observed in any cell line. The name to the left of the box is the transcription factor, and includes in parentheses the antibody used for ChIP. The letters to the right of the box indicate the cell lines in which a signal is detected, with the darkness of the letter proportional to the signal strength in the cell line. Data are derived from the Transcription Factor ChIP Track. CCCTC-binding factor (CTCF) binding was seen in multiple EBV-transformed B cell lines (G, g) as well as a variety of other cell lines. (F) CTCF binding to primary CD20+ B cells by ChIP-seq. Peak occupancy lies over exon 1 and the 5′ UTR. Data are derived from the Broad Histone Track. (G) Transcription levels for several cell types assayed by high-throughput sequencing of polyadenylated RNA (RNA-seq). Each cell line is associated with a particular colour; the GM12878 cell line is shown in pink. This figure was obtained from the UCSC Genome Browser (Human Feb 2009 (GRCh37/hg19) Assembly; http://genome.ucsc.edu).^40^

**Figure 4:**
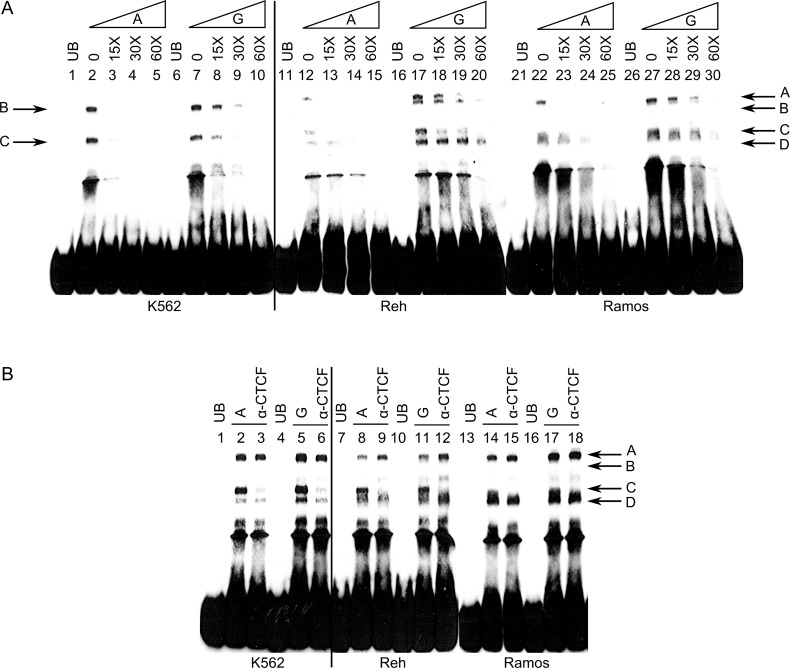
Allelic differences in complex formation at rs1876453. (A) Protein-DNA complexes (indicated by arrows; A–D) were formed with oligonucleotides including either the minor A or major G allele in the absence or presence of K562 (Lanes 1–10), Reh (Lanes 11–20) and Ramos (Lanes 21–30) nuclear extracts. Specificity and binding affinity of the protein-DNA complexes were demonstrated by the addition of 15-molar to 60-molar excess of unlabelled oligonucleotides. UB represents unbound control. (B) Anti-CTCF (CCCTC-binding factor) antibody was included during the formation of protein-DNA complexes to determine whether CTCF was involved in forming these complexes. Data shown are representative of at least three independent experiments.

**Figure 5:**
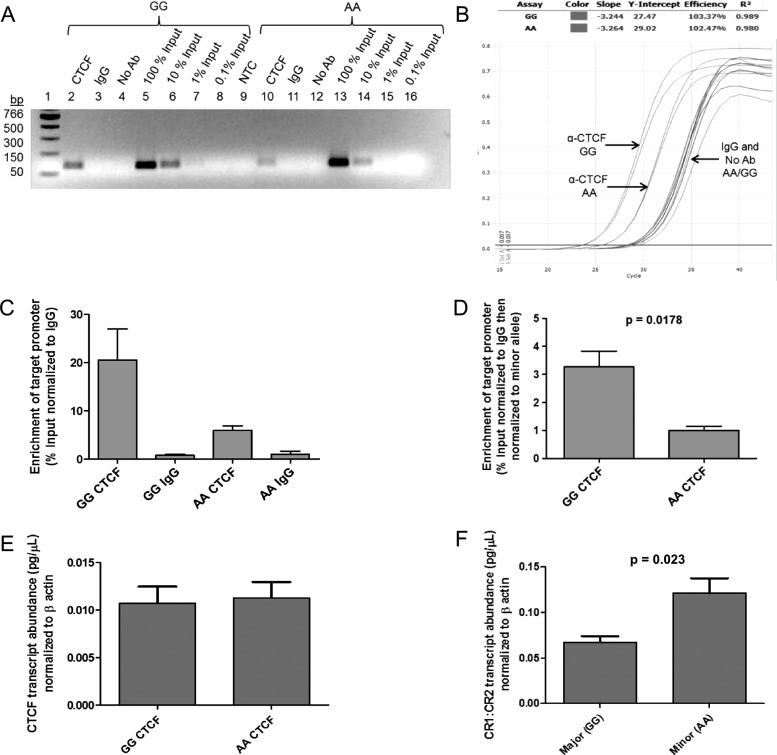
CCCTC-binding factor (CTCF) interacts with *CR2* intron 1 in vivo and demonstrates differential affinity for rs1876453 alleles. (A) Chromatin immunoprecipitation performed using an antibody specific for CTCF yielded allele-specific enrichment of the region surrounding rs1876453 from Epstein–Barr virus (EBV)-transformed B cells homozygous for the major or minor allele at rs1876453. The qPCR products were visualised by ethidium bromide staining of a 1.8% agarose gel and sized using a PCR marker (New England Biolabs). A non-specific IgG control (IgG) and a control without antibody (No Ab) were included to measure background enrichment. Decreasing amounts of enrichment were observed with serially diluted input samples (Lanes 5–8; 13–16). NTC, no template control. (B) A representative qPCR amplification plot. (C) The percentage enrichment at the *CR2* promoter was determined by quantification against the standard curve. CTCF enrichment was normalised to the background enrichment generated by a non-specific IgG. (D) CTCF enrichment normalised to the minor allele at rs1876453. (E) CTCF transcript abundance for each homozygous cell line after normalisation to β-actin. (F) Transcript abundance of *CR1* relative to *CR2* in each homozygous cell line, with each transcript normalised to β-actin. Data shown are the mean±SEM for three independent experiments.

rs1876453 is 97 nucleotides from the 5′ end of the first intron of *CR2*. This ∼12 kb intron contains a conserved silencing domain that controls *CR2* expression in a cell type-specific and developmentally regulated manner.^19–22^ The ENCyclopedia Of DNA Elements (ENCODE) database^23^ reports the in vivo interaction of the region surrounding rs1876453 with multiple transcription factors in B cells, including Pax5, Oct2 and CTCF (figure 3). We confirmed the formation of multiple protein-DNA complexes containing the sequence surrounding rs1876453 by electrophoretic mobility shift assay (EMSA) using NE from several cell lines (K562, erythroid, CR1^+^CR2^−^; Reh, pre-B, CR1^+^CR2^−^; Ramos, mature B, CR1^+^CR2^+^, see online supplementary figure S3). In the presence of the minor A allele at rs1876453, transcription factor binding was reduced or ablated and less cold competitor was required to out-compete protein-DNA complexes (figure 4A). Addition of an antibody to CTCF blocked the formation of complex C (figure 4B), and specific enrichment of the region surrounding rs1876453 was three-fold higher in the presence of the major allele by ChIP-PCR (p=0.0178; figure 5A–D), suggesting that CTCF binds the region surrounding rs1876453 in an allele-specific manner. CTCF abundance was comparable between the two cell lines (figure 5E), and transcript abundance of *CR1* relative to *CR2* was higher in the cell line homozygous for the minor allele (p=0.023; figure 5F), consistent with the data from primary B cells.

## Discussion

The minor allele of rs1876453, located in intron 1 of *CR2*, was associated with decreased risk of lupus in three of four ancestral groups studied, with the strongest association when cases were stratified based on the presence of dsDNA autoantibodies. This allele altered the formation of multiple DNA-protein complexes, including one containing CTCF, which has been termed the master regulator of chromatin organisation. Furthermore, it was associated with increased B cell expression of *CR1*, suggesting long-range effects of rs1876453 on gene regulation and providing a plausible mechanism by which it may alter lupus susceptibility.

We previously identified the SLE-associated haplotype tagged by the minor allele of rs1876453 (H1 as shown in figure 1D) in EA,^8^ and show here that it is found also in AA and HS (figure 1D). Although it is difficult to prove that a SNP is causal based on association testing, our transancestral study, which analysed a high density of SNP markers and capitalised on the weak linkage disequilibrium around rs1876453 in AA (figure 1B), provides compelling evidence that rs1876453 rather than the previously identified SNPs (rs3813946, rs1048971, rs17615 and rs4308977) or SNPs tightly linked to rs1876453 in EA (rs17258955 and rs61821130 in intron 1, rs61735651 in exon 14 and rs7549152 in intron 15) best explains the association signal. Modest association signals of rs1876453 increased after stratification of subjects based on specific clinical manifestations of lupus, with the strongest signals in the case-control analysis when subjects were stratified based on the presence of dsDNA autoantibodies. Although increased signals were seen with other clinical manifestations, only dsDNA autoantibodies were significantly associated in both case-control and case-only analyses, suggesting that rs1876453 has a preferential effect on the production or presence of dsDNA autoantibodies. The mechanism underlying this dsDNA specificity is unclear, and characterising it will lead to a greater understanding of the etiopathogenesis of lupus, since dsDNA autoantibodies are highly specific for SLE (reviewed in refs^24^ ^25^), and can predict onset and exacerbation of disease.^26–28^ Anti-dsDNA antibody levels in the serum of genotyped patients with SLE did not correlate with rs1876453 genotype (data not shown), but therapy and other disease-related factors may obscure an association. Future studies with longitudinal analyses of patients will address this more stringently.

rs1876453 is located in a transcriptional regulatory region defined by DNase hypersensitivity and containing histone marks associated with active enhancers (H3K27ac, H3K4me1 and H3K4me3) (figure 3). We demonstrated that the minor allele at rs1876453 alters the formation of multiple protein-DNA complexes (figure 4A) and that CTCF is a component of one of these complexes (figures 4B and 5). CTCF, a highly conserved and ubiquitously expressed zinc finger protein, is a master regulator of genome spatial organisation that mediates chromatin loops within the genome.^29^ By binding to insulators and boundary elements, it demarcates chromatin into regulatory regions and blocks communication between promoters and enhancers to regulate gene expression. CTCF interacted with the region surrounding rs1876453 in both lymphoblastoid B cell lines and primary B cells,^23^ with peak occupancy directly over rs1876453 (figure 3), and our data demonstrate an allele-dependent effect on its binding in vivo (figure 5) with less binding of CTCF to the minor A allele, which is associated with higher CR1 expression and is protective, and more binding to the major G risk allele, which is associated with lower CR1 expression. These data suggest that CTCF may have a repressive effect on CR1 transcription when bound to this locus. Efforts to identify the other transcription factors differentially binding this region as a result of the allele at rs1876453 are currently underway.

*CR1* expression was increased in B cells of subjects harbouring the minor allele at rs1876453 at both the mRNA and protein level (figure 2). Eukaryotic transcription is tightly controlled by regulatory elements that, though perhaps distant from their target genes, can be brought into close proximity with the general transcriptional machinery at transcription start sites through the formation of chromatin loops.^30^ Therefore, altered CTCF binding at intron 1 of *CR2* may directly affect *CR1* transcription via alterations in the chromatin looping that regulates these genetic regions. Alternatively, CR1 expression may be modified indirectly by regulatory RNA generated from intronic *CR2* sequences. The minor allele at rs1876453 introduces a cryptic splice acceptor site (consensus value (CV) 71.05, CV variation of mutant from wild type 68.75%) and is predicted to break splice enhancer and silencer motifs (Human Splicing Finder^31^). Although there are no known microRNA sequences in the first intron of *CR2*, several expressed sequence tags have been aligned to this region in B cells^32^ and the ENCODE project has determined that it is actively transcribed (figure 3).

CR1 binds C3b and C4b activation fragments and is physically associated with CR2 on B cells.^33^ Although it has an independent negative regulatory role in B cell receptor (BCR)-mediated B cell activation,^34^ it also acts as a cofactor for the Factor I-mediated cleavage of C4b to iC4b and of C3b to iC3b and C3dg. iC3b and C3dg are specific ligands for CR2^35^ that either augment or inhibit signalling through the BCR in mature B cells,^36^ ^37^ depending on ligand valency. Strength of BCR signalling may also affect tolerance induction in transitional B cells.^38^ Therefore, we hypothesise that increased expression of CR1 associated with rs1876453, in individuals susceptible to lupus, may tolerise transitional or arrest mature dsDNA-specific B cells that encounter complement-coated apoptotic debris and, as a consequence, modify the initiation or course of lupus.

In sum, we show that the minor allele at rs1876453 reduces risk of lupus and that this effect is due, in part, to increased expression of *CR1* on B cells. Since this allele alters binding of transcription factors with long-range effects, allelic variation at this SNP may modify expression of additional genes involved in lupus pathogenesis. Given the common association of rs1876453 across ancestral groups, further investigation of its mechanisms and effects has broad implications for the management of patients with this disease.

## Supplementary Material

Web figures

Web tables
