# Supplementary material for: Preferential association of a functional variant in complement receptor 2 with antibodies to double-stranded DNA
Source: Ann Rheum Dis. 2014 Sep 1;75(1):242–52. doi: 10.1136/annrheumdis-2014-205584 (PMC4717392; doi:10.1136/annrheumdis-2014-205584)
Supplement: Web figures [file annrheumdis-2014-205584-s1.pdf]

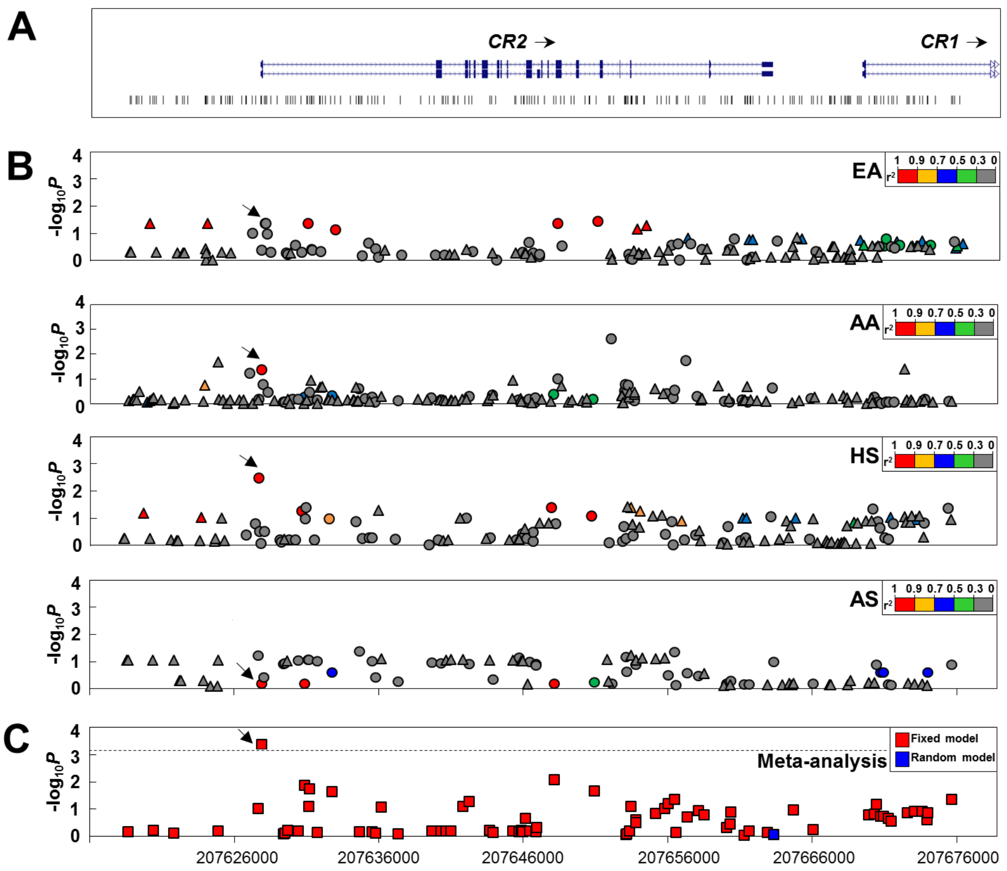

Figure S1. Association of SNPs in the *CR2* region with SLE. (A) The genomic structure of the *CR2* region and positions of genetic variants are indicated. (B) The allelic *P* value ($-\log_{10}P$ value) of each genetic variant with SLE is plotted against its position as a circle (genotyped) or a triangle (imputed) for European American (EA), African American (AA), Hispanic (HS) and Asian (AS), respectively. Genetic variants are highlighted using different colors according to their LD strength (r^2) with rs1876453. An arrow is used to indicate the position of rs1876453. (C) Trans-ancestral meta-analysis *P* value generated using fixed and random model are highlighted as red and blue, respectively. The dashed line represents the significance level after Bonferroni correction.

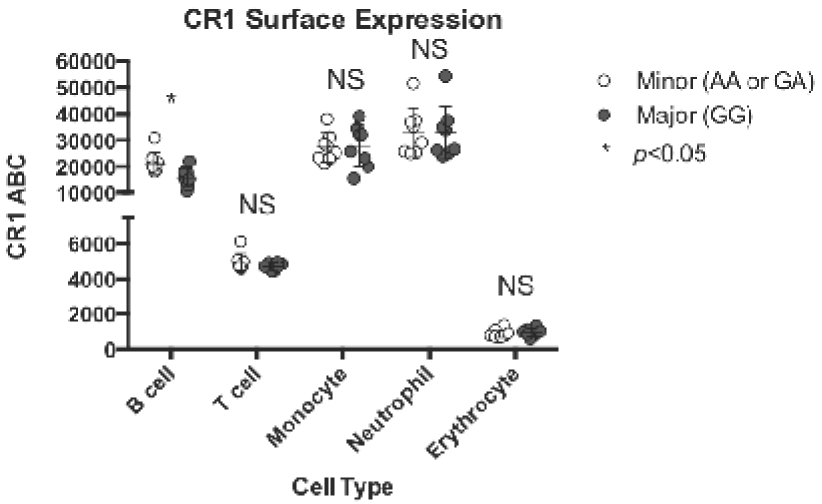

Figure S2. Expression of CR1 on peripheral blood cells. Peripheral blood cell subsets were identified by flow cytometry using fluorescent antibodies for CD19, CD3, and CD235 as well as forward and side scatter characteristics. CR1 levels were determined using quantitative microbeads. Subjects were also stratified based on whether they had the high (H) or low (L) expression alleles for CR1 on erythrocytes, which is determined by a HindIII restriction fragment length polymorphism (J Exp Med 164: 50-59, 1986; data not shown). Allele-specific differences in CR1 expression were only seen in B cells. Mean ± SD is shown. ABC, antibody binding capacity.

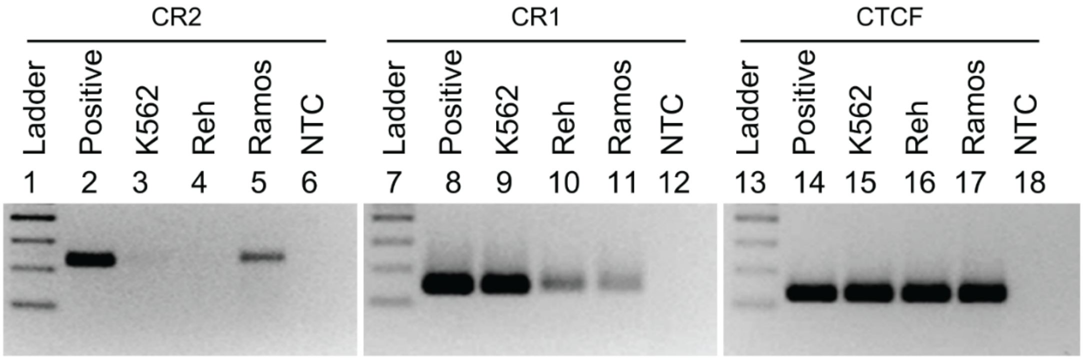

Figure S3. ***CR1*** **and** ***CR2*** **transcription in cell lines used for EMSA.** To determine the gene expression status of the cell lines K562 (erythroid), Reh (pre-B) and Ramos (mature B) used for EMSA, rtPCR was performed with primers specific for *CR2* (lanes 2 – 6), *CR1* (lanes 8 – 12) or *CTCF* (lanes 14 – 18). Based on the presence/absence of a specific PCR amplicon, gene expression was determined as follows; K562 ($CR2^-$ lane 3, $CR1^+$ lane 9, $CTCF^+$ lane 15), Reh ($CR2^-$ lane 4, $CR1^+$ lane 10, $CTCF^+$ lane 16) and Ramos ($CR2^+$ lane 5, $CR1^+$ lane 11, $CTCF^+$ lane 17). A purified cDNA sample of each gene of interest (positive control, lanes 2, 8, 14) and a no template control (NTC, lanes 6, 12, 18) was included for each primer set. PCR products were electrophoresed on a 1.5% agarose gel strained with ethidium bromide for visualization. Amplicons were compared to a molecular ladder of known size standards (100 bp ladder, New England Biolabs, Ipswich, MA, USA).
