# Supplementary material for: Preferential association of a functional variant in complement receptor 2 with antibodies to double-stranded DNA
Source: Ann Rheum Dis. 2014 Sep 1;75(1):242–52. doi: 10.1136/annrheumdis-2014-205584 (PMC4717392; doi:10.1136/annrheumdis-2014-205584)
Supplement: Web tables [file annrheumdis-2014-205584-s2.pdf]

Table S1. Association of *CR2* variants with SLE in multiple ancestral groups

						EA				AA				AS				HS				Meta	
						Freq				Freq				Freq				Freq					
Annotation	Type	SNP	Position	A1	A2	SLE	CTRL	*P*	OR [95%CI]	SLE	CTRL	*P*	OR[95%CI]	SLE	CTRL	*P*	OR[95%CI]	SLE	CTRL	*P*	OR[95%CI]	*P*	OR
CR2 5' upstream	I	rs311321	207618591	A	G	29.79%	30.57%	0.47	0.97[0.90-1.05]	--	--	--	--	12.36%	10.85%	0.093	1.16[0.98-1.38]	21.03%	21.15%	0.55	1.05[0.90-1.22]	--	--
CR2 5' upstream	I	rs311320	207618661	G	T	29.93%	30.62%	0.51	0.97[0.90-1.05]	43.78%	43.04%	0.77	1.02[0.92-1.12]	12.36%	10.85%	0.093	1.16[0.98-1.38]	21.32%	21.49%	0.57	1.05[0.90-1.22]	0.68	1.01
CR2 5' upstream	I	rs73089144	207618967	G	A	--	--	--	--	8.37%	8.83%	0.66	0.96[0.81-1.15]	--	--	--	--	--	--	--	--	--	--
CR2 5' upstream	I	rs59808407	207619051	T	C	--	--	--	--	8.37%	8.83%	0.66	0.96[0.81-1.15]	--	--	--	--	--	--	--	--	--	--
CR2 5' upstream	I	rs138684626	207619203	G	A	--	--	--	--	2.64%	2.84%	0.74	0.95[0.71-1.28]	--	--	--	--	--	--	--	--	--	--
CR2 5' upstream	I	rs57767408	207619399	C	T	--	--	--	--	3.24%	2.69%	0.34	1.15[0.86-1.53]	--	--	--	--	--	--	--	--	--	--
CR2 5' upstream	I	rs17044032	207619949	G	A	8.77%	9.93%	***0.045***	0.89[0.79-0.99]	10.86%	10.67%	0.88	0.99[0.84-1.16]	--	--	--	--	4.27%	5.62%	0.067	0.76[0.57-1.02]	--	--
CR2 5' upstream	I	rs114928704	207620136	A	C	--	--	--	--	4.23%	4.17%	0.76	0.96[0.76-1.22]	--	--	--	--	--	--	--	--	--	--
CR2 5' upstream	I	rs12081383	207620289	C	A	--	--	--	--	8.37%	8.83%	0.66	0.96[0.81-1.15]	--	--	--	--	--	--	--	--	--	--
CR2 5' upstream	I	rs311319	207620401	G	A	29.69%	30.42%	0.51	0.98[0.91-1.05]	43.11%	42.19%	0.61	1.03[0.93-1.13]	12.36%	10.85%	0.093	1.16[0.98-1.38]	21.03%	21.15%	0.55	1.05[0.90-1.22]	0.60	1.01
CR2 5' upstream	I	rs148632661	207620900	G	T	--	--	--	--	2.64%	2.84%	0.74	0.95[0.71-1.28]	--	--	--	--	--	--	--	--	--	--
CR2 5' upstream	I	rs184612141	207621755	G	A	--	--	--	--	1.50%	1.48%	1.00	1.00[0.67-1.50]	--	--	--	--	--	--	--	--	--	--
CR2 5' upstream	I	rs311317	207621832	A	G	29.81%	30.47%	0.54	0.98[0.91-1.05]	53.08%	52.70%	0.90	1.01[0.91-1.11]	12.36%	10.85%	0.093	1.16[0.98-1.38]	21.90%	22.11%	0.62	1.04[0.89-1.21]	0.75	1.01
CR2 5' upstream	I	rs311316	207622223	A	T	12.04%	11.51%	0.53	1.04[0.93-1.15]	--	--	--	--	8.80%	9.25%	0.53	0.94[0.78-1.14]	9.71%	10.24%	0.66	0.95[0.78-1.17]	--	--
CR2 5' upstream	I	rs311315	207622318	G	T	12.04%	11.51%	0.53	1.04[0.93-1.15]	--	--	--	--	8.80%	9.25%	0.53	0.94[0.78-1.14]	9.71%	10.24%	0.66	0.95[0.78-1.17]	--	--
CR2 5' upstream	I	rs191006951	207622587	T	C	--	--	--	--	1.32%	1.38%	0.72	0.93[0.61-1.41]	--	--	--	--	--	--	--	--	--	--
CR2 5' upstream	I	rs17044219	207623781	A	G	1.24%	1.09%	0.39	1.15[0.84-1.59]	--	--	--	--	--	--	--	--	--	--	--	--	--	--
CR2 5' upstream	I	rs7527218	207623829	G	A	46.81%	46.66%	0.99	1.00[0.93-1.07]	72.93%	73.21%	0.85	0.99[0.89-1.11]	--	--	--	--	--	--	--	--	--	--
CR2 5' upstream	I	rs311314	207623868	G	A	12.04%	11.51%	0.53	1.04[0.93-1.15]	--	--	--	--	8.80%	9.25%	0.53	0.94[0.78-1.14]	9.71%	10.24%	0.66	0.95[0.78-1.17]	--	--
CR2 5' upstream	I	rs61240730	207623930	C	T	8.77%	9.93%	***0.045***	0.89[0.79-0.99]	5.84%	6.40%	0.19	0.87[0.71-1.07]	--	--	--	--	4.27%	5.51%	0.10	0.78[0.59-1.05]	--	--
CR2 5' upstream	I	rs71635139	207624289	A	T	7.02%	7.25%	0.98	1.00[0.87-1.14]	--	--	--	--	--	--	--	--	--	--	--	--	--	--
CR2 5' upstream	I	rs72644189	207624395	T	C	--	--	--	--	--	--	--	--	27.13%	27.39%	0.85	0.99[0.87-1.12]	--	--	--	--	--	--
CR2 5' upstream	I	rs11808746	207624861	T	A	--	--	--	--	2.58%	1.74%	***0.021***	1.49[1.06-2.09]	27.13%	27.39%	0.85	0.99[0.87-1.12]	1.05%	1.26%	0.73	0.90[0.51-1.61]	--	--
CR2 5' upstream	I	rs311313	207624911	C	T	29.69%	30.42%	0.51	0.98[0.91-1.05]	42.89%	42.13%	0.67	1.02[0.93-1.13]	12.36%	10.85%	0.093	1.16[0.98-1.38]	21.03%	21.15%	0.55	1.05[0.90-1.22]	0.63	1.01
CR2 5' upstream	I	rs188721882	207625219	C	T	--	--	--	--	1.32%	1.38%	0.72	0.93[0.61-1.41]	--	--	--	--	--	--	--	--	--	--
CR2 5' upstream	I	1-207625357	207625357	TAA	T	--	--	--	--	--	--	--	--	--	--	--	--	6.28%	5.29%	0.10	1.26[0.96-1.65]	--	--
CR2 5' upstream	I	rs143193885	207625498	G	A	--	--	--	--	1.50%	1.48%	1.00	1.00[0.67-1.50]	--	--	--	--	--	--	--	--	--	--
CR2 5' upstream	I	rs311312	207625541	G	T	12.04%	11.51%	0.53	1.04[0.93-1.15]	--	--	--	--	--	--	--	--	9.71%	10.24%	0.66	0.95[0.78-1.17]	--	--
CR2 5' upstream	I	rs144800606	207625690	C	T	--	--	--	--	1.32%	1.38%	0.72	0.93[0.61-1.41]	--	--	--	--	--	--	--	--	--	--
CR2 5' upstream	I	rs151025105	207626329	A	T	--	--	--	--	1.32%	1.38%	0.72	0.93[0.61-1.41]	--	--	--	--	--	--	--	--	--	--
CR2 5' upstream	I	rs140024463	207626613	A	C	--	--	--	--	1.50%	1.48%	1.00	1.00[0.67-1.50]	--	--	--	--	--	--	--	--	--	--
CR2 5' upstream	I	rs143641831	207626646	A	G	--	--	--	--	1.32%	1.38%	0.72	0.93[0.61-1.41]	--	--	--	--	--	--	--	--	--	--
CR2 5' upstream	G	rs12135588	207627057	G	A	2.36%	2.91%	0.10	0.83[0.67-1.04]	0.57%	0.29%	0.061	2.12[0.97-4.66]	0.00%	0.00%	ND	ND	0.90%	1.22%	0.42	0.78[0.43-1.43]	--	--
CR2 5' upstream	I	rs140630338	207627293	G	T	--	--	--	--	1.50%	1.48%	1.00	1.00[0.67-1.50]	--	--	--	--	--	--	--	--	--	--
CR2 5'UTR	I	rs182309299	207627672	T	C	--	--	--	--	2.64%	2.84%	0.74	0.95[0.71-1.28]	--	--	--	--	--	--	--	--	--	--
CR2 5'UTR	G	rs3813946	207627693	C	T	19.72%	19.22%	0.42	1.04[0.95-1.13]	7.98%	8.20%	0.67	0.96[0.80-1.15]	11.91%	10.25%	0.064	1.19[0.99-1.42]	13.58%	12.84%	0.16	1.14[0.95-1.38]	0.096	1.06
CR2 intron1	G	rs1876453	207627918	A	G	8.77%	9.93%	***0.045***	0.89[0.79-0.99]	7.48%	8.56%	***0.045***	0.83[0.70-0.99]	0.04%	0.08%	0.68	0.60[0.05-6.74]	3.18%	5.10%	***3.5E-03***	0.62[0.46-0.86]	***4.2E-04***	0.85
CR2 intron1	G	rs17258955	207627998	T	C	3.37%	4.02%	***0.044***	0.83[0.69-0.99]	0.75%	0.52%	0.18	1.54[0.82-2.87]	0.00%	0.00%	ND	ND	1.32%	1.83%	0.31	0.77[0.47-1.27]	--	--
CR2 intron1	G	rs41304115	207628076	A	G	2.87%	3.26%	0.11	0.85[0.69-1.04]	--	--	--	--	0.04%	0.12%	0.39	0.37[0.04-3.66]	1.35%	1.43%	0.86	1.05[0.62-1.76]	--	--
CR2 intron1	G	rs41304111	207628347	G	A	2.36%	2.19%	0.50	1.08[0.86-1.36]	0.45%	0.39%	0.35	1.44[0.67-3.11]	0.00%	0.00%	ND	ND	1.43%	1.13%	0.31	1.34[0.76-2.35]	--	--
CR2 intron1	I	rs116762805	207628788	T	A	--	--	--	--	4.23%	4.17%	0.76	0.96[0.76-1.22]	--	--	--	--	--	--	--	--	--	--
CR2 intron1	G	rs311302	207629416	T	C	30.03%	30.74%	0.53	0.98[0.91-1.05]	44.47%	44.00%	0.90	1.01[0.91-1.11]	12.37%	10.99%	0.13	1.14[0.96-1.36]	21.53%	21.84%	0.66	1.04[0.89-1.20]	0.81	1.01
CR2 intron1	G	rs311303	207629514	G	A	30.12%	30.65%	0.62	0.98[0.91-1.06]	53.04%	52.65%	0.88	1.01[0.92-1.11]	12.36%	10.96%	0.12	1.15[0.96-1.36]	22.18%	22.68%	0.78	1.02[0.88-1.18]	0.75	1.01
CR2 intron1	I	rs311304	207629702	A	C	29.81%	30.48%	0.55	0.98[0.91-1.05]	42.89%	42.13%	0.67	1.02[0.93-1.13]	12.36%	10.88%	0.10	1.16[0.97-1.37]	21.10%	21.30%	0.59	1.04[0.90-1.21]	0.62	1.01
CR2 intron1	I	rs146939930	207629863	G	A	--	--	--	--	1.50%	1.48%	1.00	1.00[0.67-1.50]	--	--	--	--	--	--	--	--	--	--
CR2 intron1	G	rs311305	207630032	G	A	0.05%	0.07%	0.28	0.47[0.12-1.85]	11.53%	11.24%	0.69	1.03[0.88-1.20]	0.00%	0.00%	ND	ND	0.64%	0.69%	0.65	0.84[0.39-1.78]	--	--
CR2 intron1	G	rs311306	207630444	C	G	29.70%	30.42%	0.51	0.98[0.91-1.05]	42.87%	42.08%	0.66	1.02[0.93-1.13]	12.36%	10.85%	0.095	1.16[0.98-1.38]	21.14%	21.38%	0.63	1.04[0.89-1.21]	0.65	1.01
CR2 intron1	I	rs150868219	207630836	T	C	--	--	--	--	1.50%	1.48%	1.00	1.00[0.67-1.50]	--	--	--	--	--	--	--	--	--	--
CR2 intron1	G	rs61821130	207630888	A	C	8.68%	9.88%	***0.044***	0.89[0.79-0.99]	12.05%	12.16%	0.55	0.96[0.83-1.11]	0.04%	0.08%	0.68	0.60[0.05-6.73]	4.54%	5.95%	0.057	0.76[0.58-1.01]	***0.014***	0.90
CR2 intron1	I	rs73089153	207631048	A	T	--	--	--	--	8.53%	8.89%	0.71	0.97[0.81-1.15]	--	--	--	--	--	--	--	--	--	--
CR2 intron1	G	rs12140602	207631158	A	T	20.99%	20.45%	0.41	1.04[0.95-1.13]	9.22%	9.41%	0.77	0.98[0.83-1.15]	12.32%	10.80%	0.092	1.16[0.98-1.38]	14.93%	13.97%	0.11	1.15[0.97-1.37]	0.083	1.06
CR2 intron1	G	rs12139795	207631205	G	A	20.97%	20.43%	0.41	1.04[0.95-1.13]	16.51%	15.72%	0.36	1.06[0.93-1.21]	12.33%	10.80%	0.090	1.16[0.98-1.38]	15.67%	14.12%	***0.041***	1.20[1.01-1.42]	***0.019***	1.08
CR2 intron1	I	rs80182520	207631332	T	A	--	--	--	--	7.36%	6.41%	0.11	1.17[0.96-1.41]	--	--	--	--	--	--	--	--	--	--
CR2 intron1	I	rs146796037	207631753	T	C	--	--	--	--	1.50%	1.48%	1.00	1.00[0.67-1.49]	--	--	--	--	--	--	--	--	--	--
CR2 intron1	G	rs311307	207631778	T	C	29.79%	30.55%	0.49	0.97[0.90-1.05]	42.72%	42.09%	0.74	1.02[0.92-1.12]	12.36%	10.88%	0.10	1.16[0.97-1.37]	21.23%	21.49%	0.64	1.04[0.89-1.21]	0.72	1.01
CR2 intron1	I	rs77276388	207631951	C	T	--	--	--	--	4.08%	3.41%	0.22	1.17[0.91-1.51]	--	--	--	--	--	--	--	--	--	--
CR2 intron1	I	rs77625468	207632129	C	G	--	--	--	--	2.73%	2.32%	0.21	1.22[0.89-1.67]	--	--	--	--	--	--	--	--	--	--
CR2 intron1	I	rs311308	207632487	A	G	--	--	--	--	11.57%	11.28%	0.66	1.04[0.89-1.21]	--	--	--	--	--	--	--	--	--	--
CR2 intron1	I	rs142880184	207632526	C	G	--	--	--	--	1.32%	1.38%	0.72	0.93[0.61-1.41]	--	--	--	--	--	--	--	--	--	--
CR2 intron1	G	rs17044525	207632796	G	A	8.83%	9.89%	0.077	0.90[0.80-1.01]	13.51%	13.64%	0.49	0.95[0.83-1.10]	0.04%	0.16%	0.27	0.29[0.03-2.61]	4.86%	6.15%	0.11	0.80[0.61-1.05]	***0.024***	0.91
CR2 intron1	I	rs150973349	207632805	C	T	--	--	--	--	1.32%	1.38%	0.72	0.93[0.61-1.41]	--	--	--	--	--	--	--	--	--	--
CR2 intron1	I	rs116542838	207633194	C	T	--	--	--	--	4.23%	4.17%	0.76	0.96[0.76-1.22]	--	--	--	--	--	--	--	--	--	--
CR2 intron1	I	rs146923426	207633875	G	A	--	--	--	--	1.32%	1.38%	0.72	0.93[0.61-1.41]	--	--	--	--	--	--	--	--	--	--
CR2 intron1	I	rs149048095	207634274	C	T	--	--	--	--	1.32%	1.38%	0.72	0.93[0.61-1.41]	--	--	--	--	--	--	--	--	--	--
CR2 intron1	I	rs143079233	207634294	G	A	--	--	--	--	1.32%	1.38%	0.72	0.93[0.61-1.41]	--	--	--	--	--	--	--	--	--	--
CR2 intron1	I	1-207634463	207634463	G	GT	--	--	--	--	4.08%	3.41%	0.22	1.17[0.91-1.51]	--	--	--	--	--	--	--	--	--	--
CR2 intron1	I	rs141941832	207634483	T	G	--	--	--	--	1.32%	1.35%	0.81	0.95[0.62-1.45]	--	--	--	--	--	--	--	--	--	--
CR2 intron1	I	rs74857976	207634617	G	A	--	--	--	--	4.23%	4.17%	0.76	0.96[0.76-1.22]	--	--	--	--	--	--	--	--	--	--
CR2 intron1	G	rs17044576	207634670	C	T	4.67%	4.40%	0.69	1.03[0.88-1.22]	11.97%	11.54%	0.26	1.09[0.94-1.27]	9.14%	10.88%	***0.043***	0.83[0.69-0.99]	6.14%	5.20%	0.13	1.23[0.94-1.62]	0.69	1.02
CR2 intron1	G	rs311309	207635087	G	A	0.05%	0.09%	0.23	0.44[0.12-1.67]	11.63%	11.30%	0.62	1.04[0.89-1.21]	0.00%	0.00%	ND	ND	0.68%	0.75%	0.58	0.81[0.39-1.69]	--	--

CR2 intron1	I	rs146134620	207635390	A	G	--	--	--	--	1.32%	1.35%	0.81	0.95[0.62-1.45]	--	--	--	--	--	--	--	--	--	--
CR2 intron1	G	rs311310	207635573	A	G	29.45%	30.35%	0.37	0.97[0.90-1.04]	40.38%	39.39%	0.57	1.03[0.93-1.13]	12.33%	10.85%	0.10	1.16[0.97-1.37]	20.57%	20.73%	0.53	1.05[0.90-1.22]	0.69	1.01
CR2 intron1	G	rs1567190	207635789	T	C	49.39%	49.45%	0.76	0.99[0.92-1.06]	75.97%	75.83%	0.87	1.01[0.90-1.13]	58.22%	59.35%	0.40	0.95[0.85-1.07]	40.13%	40.42%	0.55	1.04[0.92-1.18]	0.78	0.99
CR2 intron1	I	rs3767934	207636208	T	G	20.87%	20.31%	0.43	1.03[0.95-1.13]	7.86%	8.28%	0.52	0.94[0.79-1.13]	12.32%	10.77%	0.085	1.16[0.98-1.38]	14.63%	13.34%	0.052	1.19[1.00-1.42]	0.084	1.06
CR2 intron1	G	rs3767933	207637359	T	C	49.45%	49.63%	0.65	0.98[0.92-1.05]	76.11%	75.83%	0.76	1.02[0.91-1.14]	58.69%	59.42%	0.57	0.97[0.86-1.08]	39.92%	40.32%	0.59	1.04[0.91-1.17]	0.82	0.99
CR2 intron1	I	1-207638763	207638763	T	TA	--	--	--	--	3.82%	4.04%	0.67	0.95[0.74-1.22]	--	--	--	--	--	--	--	--	--	--
CR2 intron1	I	rs115281878	207639384	G	A	--	--	--	--	3.82%	4.02%	0.72	0.95[0.74-1.23]	--	--	--	--	--	--	--	--	--	--
CR2 intron1	G	rs311311	207639695	C	G	29.98%	30.50%	0.64	0.98[0.91-1.06]	52.07%	51.30%	0.60	1.03[0.93-1.13]	12.36%	10.93%	0.11	1.15[0.97-1.36]	22.11%	22.86%	0.96	1.00[0.87-1.16]	0.65	1.01
CR2 intron2	I	rs145095346	207640274	G	A	--	--	--	--	3.82%	4.00%	0.73	0.96[0.75-1.23]	--	--	--	--	--	--	--	--	--	--
CR2 intron2	G	rs1032980	207640345	G	T	30.09%	30.63%	0.62	0.98[0.91-1.06]	44.15%	43.44%	0.74	1.02[0.92-1.12]	12.36%	10.96%	0.12	1.15[0.97-1.36]	21.53%	21.80%	0.63	1.04[0.89-1.21]	0.64	1.01
CR2 intron2	I	rs1257562	207640492	C	T	--	--	--	--	11.66%	11.41%	0.73	1.03[0.88-1.20]	--	--	--	--	--	--	--	--	--	--
CR2 intron2	I	rs1270549	207640673	A	C	29.94%	30.53%	0.59	0.98[0.91-1.06]	44.11%	43.43%	0.75	1.02[0.92-1.12]	12.36%	10.85%	0.10	1.16[0.97-1.37]	21.22%	21.63%	0.69	1.03[0.89-1.20]	0.66	1.01
CR2 intron2	I	rs1032979	207641023	C	T	29.94%	30.53%	0.59	0.98[0.91-1.06]	43.09%	42.36%	0.73	1.02[0.92-1.12]	12.36%	10.82%	0.090	1.16[0.98-1.38]	21.20%	21.53%	0.65	1.04[0.89-1.20]	0.63	1.01
CR2 intron2	I	rs17045028	207641114	G	A	--	--	--	--	3.82%	3.97%	0.75	0.96[0.75-1.23]	--	--	--	--	--	--	--	--	--	--
CR2 intron2	I	1-207641329	207641329	T	TTAAG	--	--	--	--	6.95%	7.20%	0.75	0.97[0.80-1.17]	--	--	--	--	--	--	--	--	--	--
CR2 intron2	I	rs2063143	207641840	T	C	20.90%	20.32%	0.39	1.04[0.95-1.13]	8.25%	8.49%	0.69	0.96[0.81-1.15]	12.32%	10.80%	0.092	1.16[0.98-1.38]	14.62%	13.67%	0.10	1.16[0.97-1.38]	0.080	1.06
CR2 intron3	I	rs17045039	207642125	T	A	--	--	--	--	3.71%	3.92%	0.68	0.95[0.74-1.22]	--	--	--	--	--	--	--	--	--	--
CR2 intron4	G	rs1507764	207642295	A	G	20.86%	20.46%	0.47	1.03[0.95-1.12]	15.61%	15.06%	0.54	1.04[0.91-1.19]	12.20%	10.82%	0.13	1.14[0.96-1.36]	15.52%	14.39%	0.10	1.16[0.97-1.37]	0.053	1.06
CR2 intron6	I	rs11117872	207643566	C	T	--	--	--	--	5.88%	5.85%	0.75	0.97[0.79-1.19]	--	--	--	--	--	--	--	--	--	--
CR2 intron6	I	rs568515	207643673	C	T	29.78%	30.47%	0.53	0.98[0.91-1.05]	38.92%	38.03%	0.64	1.02[0.93-1.13]	12.36%	10.85%	0.093	1.16[0.98-1.38]	20.60%	20.83%	0.58	1.04[0.90-1.22]	0.60	1.01
CR2 intron6	G	rs10863322	207643949	G	A	46.96%	46.81%	0.97	1.00[0.94-1.07]	71.73%	72.47%	0.47	0.96[0.86-1.07]	30.88%	31.75%	0.49	0.96[0.85-1.08]	38.88%	39.10%	0.54	1.04[0.92-1.18]	0.73	0.99
CR2 intron6	I	rs17045132	207643983	A	G	--	--	--	--	3.40%	3.75%	0.47	0.91[0.70-1.18]	--	--	--	--	--	--	--	--	--	--
CR2 intron9	I	rs511678	207645282	C	G	29.79%	30.46%	0.54	0.98[0.91-1.05]	38.92%	38.04%	0.65	1.02[0.93-1.13]	12.36%	10.85%	0.093	1.16[0.98-1.38]	20.58%	20.94%	0.65	1.04[0.89-1.21]	0.63	1.01
CR2 intron9	I	rs116421473	207645361	A	G	--	--	--	--	7.29%	6.35%	0.11	1.17[0.96-1.42]	--	--	--	--	--	--	--	--	--	--
CR2 intron9	G	rs2940253	207645743	C	G	29.91%	30.62%	0.49	0.97[0.90-1.05]	38.82%	38.04%	0.71	1.02[0.92-1.12]	12.44%	10.81%	0.073	1.17[0.99-1.39]	20.76%	21.03%	0.65	1.04[0.89-1.21]	0.67	1.01
CR2 intron9	I	rs9429774	207645895	A	G	29.78%	30.46%	0.53	0.98[0.91-1.05]	38.93%	38.02%	0.63	1.03[0.93-1.13]	12.33%	10.85%	0.10	1.16[0.97-1.37]	20.66%	20.94%	0.61	1.04[0.89-1.21]	0.62	1.01
CR2 intron9	I	rs9429936	207645956	A	C	29.91%	30.52%	0.58	0.98[0.91-1.06]	42.10%	41.50%	0.82	1.01[0.92-1.12]	12.33%	10.85%	0.10	1.16[0.97-1.37]	21.24%	21.46%	0.59	1.04[0.90-1.21]	0.68	1.01
CR2 exon10	G	rs34349246	207646163	T	C	1.61%	1.36%	0.21	1.20[0.90-1.60]	3.14%	3.22%	0.88	1.02[0.78-1.34]	0.04%	0.00%	ND	ND	1.80%	1.57%	0.38	1.24[0.77-2.02]	0.22	1.12
CR2 exon10	I	rs1048971	207646322	A	G	34.61%	34.92%	0.76	0.99[0.92-1.06]	--	--	--	--	21.73%	22.18%	0.72	0.98[0.85-1.11]	27.97%	27.44%	0.25	1.09[0.94-1.25]	--	--
CR2 exon10	G	rs17615	207646462	A	G	29.84%	30.53%	0.53	0.98[0.91-1.05]	38.93%	38.08%	0.66	1.02[0.93-1.13]	12.36%	10.89%	0.10	1.15[0.97-1.37]	20.91%	21.07%	0.57	1.05[0.90-1.22]	0.63	1.01
CR2 intron10	I	rs148404045	207646661	A	T	--	--	--	--	2.49%	2.62%	0.92	0.98[0.72-1.34]	--	--	--	--	--	--	--	--	--	--
CR2 exon11	G	rs4308977	207646898	C	T	29.99%	30.41%	0.71	0.99[0.92-1.06]	49.76%	49.38%	0.89	1.01[0.91-1.11]	12.33%	10.96%	0.13	1.14[0.96-1.36]	22.04%	22.42%	0.71	1.03[0.89-1.19]	0.68	1.01
CR2 exon11	G	rs17616	207646923	A	G	29.69%	30.28%	0.61	0.98[0.91-1.06]	38.60%	37.63%	0.59	1.03[0.93-1.13]	12.18%	10.87%	0.15	1.14[0.96-1.35]	20.28%	19.95%	0.34	1.08[0.92-1.26]	0.49	1.02
CR2 intron12	I	rs9429775	207647339	G	A	--	--	--	--	22.51%	22.97%	0.67	0.97[0.87-1.10]	--	--	--	--	1.55%	2.03%	0.16	0.71[0.44-1.14]	--	--
CR2 intron13	I	rs3991865	207647993	A	C	--	--	--	--	21.79%	22.03%	0.84	0.99[0.88-1.11]	--	--	--	--	1.55%	2.03%	0.16	0.71[0.44-1.14]	--	--
CR2 exon14	G	rs61735651	207648173	C	T	8.47%	9.60%	***0.043***	0.89[0.79-0.99]	10.89%	11.22%	0.43	0.94[0.81-1.10]	0.04%	0.08%	0.68	0.60[0.05-6.74]	4.36%	5.79%	***0.041***	0.74[0.56-0.99]	***8.6E-03***	0.89
CR2 exon14	G	rs1143665	207648494	C	T	0.05%	0.10%	0.30	0.50[0.14-1.84]	7.31%	6.35%	0.10	1.17[0.97-1.42]	0.00%	0.00%	ND	ND	0.78%	0.38%	0.17	1.90[0.76-4.72]	--	--
CR2 intron14	I	1-207648720	207648720	A	ATTGT	--	--	--	--	4.04%	3.34%	0.20	1.19[0.92-1.53]	--	--	--	--	--	--	--	--	--	--
CR2 intron14	I	rs9429776	207649224	A	G	--	--	--	--	18.27%	18.18%	0.89	1.01[0.89-1.15]	--	--	--	--	--	--	--	--	--	--
CR2 intron14	I	rs143926411	207649434	C	G	--	--	--	--	1.50%	1.41%	0.73	1.07[0.71-1.61]	--	--	--	--	--	--	--	--	--	--
CR2 intron15	I	rs9429777	207650191	G	A	--	--	--	--	11.78%	11.51%	0.75	1.03[0.88-1.19]	--	--	--	--	--	--	--	--	--	--
CR2 intron15	I	rs9429938	207650464	C	A	--	--	--	--	18.12%	17.99%	0.88	1.01[0.89-1.15]	--	--	--	--	--	--	--	--	--	--
CR2 intron15	I	rs150797291	207650498	G	T	--	--	--	--	3.49%	3.75%	0.63	0.94[0.72-1.22]	--	--	--	--	--	--	--	--	--	--
CR2 intron15	G	rs7549152	207650938	T	G	8.56%	9.81%	***0.037***	0.88[0.79-0.99]	11.36%	11.44%	0.69	0.97[0.83-1.13]	0.12%	0.08%	0.59	1.54[0.33-7.25]	4.30%	5.57%	0.086	0.78[0.58-1.04]	***0.022***	0.90
CR2 intron16	I	rs9429778	207651835	A	C	29.78%	30.41%	0.57	0.98[0.91-1.05]	--	--	--	--	12.23%	10.71%	0.090	1.16[0.98-1.38]	--	--	--	--	--	--
CR2 intron16	I	rs4844596	207651935	A	G	46.65%	46.45%	0.92	1.00[0.94-1.08]	--	--	--	--	30.91%	31.61%	0.58	0.97[0.86-1.09]	--	--	--	--	--	--
CR2 intron16	G	rs17045328	207652176	G	A	--	--	--	--	3.17%	2.06%	***0.0026***	1.61[1.18-2.20]	26.70%	27.24%	0.68	0.97[0.86-1.11]	1.16%	1.33%	0.79	0.93[0.53-1.62]	--	--
CR2 intron17	I	rs147029386	207652928	C	A	--	--	--	--	3.36%	3.74%	0.47	0.91[0.70-1.18]	--	--	--	--	--	--	--	--	--	--
CR2 intron17	I	rs138313826	207652934	T	C	--	--	--	--	3.37%	3.73%	0.47	0.91[0.70-1.18]	--	--	--	--	--	--	--	--	--	--
CR2 intron17	I	rs1475445	207652987	G	T	--	--	--	--	11.15%	11.05%	0.97	1.00[0.86-1.17]	--	--	--	--	--	--	--	--	--	--
CR2 intron17	I	rs4317805	207652992	A	G	--	--	--	--	1.08%	1.15%	0.92	1.02[0.64-1.63]	--	--	--	--	--	--	--	--	--	--
CR2 intron17	G	rs2182909	207653106	C	A	29.04%	29.74%	0.59	0.98[0.91-1.06]	21.41%	22.00%	0.30	0.94[0.83-1.06]	12.27%	10.66%	0.071	1.17[0.99-1.40]	19.03%	19.51%	0.72	1.03[0.88-1.20]	0.86	0.99
CR2 intron17	G	rs12032512	207653178	G	C	46.23%	46.84%	0.84	0.99[0.93-1.06]	24.27%	25.13%	0.17	0.92[0.83-1.03]	44.29%	42.76%	0.25	1.07[0.96-1.19]	41.76%	39.80%	0.21	1.08[0.96-1.23]	0.80	1.01
CR2 exon18	G	rs17258982	207653364	G	A	7.83%	7.75%	0.91	1.01[0.89-1.14]	8.95%	9.75%	0.19	0.90[0.76-1.05]	0.00%	0.16%	ND	ND	5.15%	5.14%	0.55	1.09[0.83-1.43]	0.63	0.98
CR2 exon18	I	rs6540433	207653395	C	A	--	--	--	--	1.93%	2.07%	0.54	0.90[0.63-1.27]	--	--	--	--	--	--	--	--	--	--
CR2 intron18	I	rs17258996	207653444	A	T	20.85%	20.34%	0.44	1.03[0.95-1.12]	6.50%	6.99%	0.34	0.91[0.75-1.11]	12.39%	10.74%	0.064	1.18[0.99-1.40]	14.55%	13.11%	***0.038***	1.21[1.01-1.44]	0.082	1.06
CR2 intron18	I	rs7548495	207653701	A	T	8.39%	9.45%	0.069	0.90[0.80-1.01]	--	--	--	--	--	--	--	--	3.83%	5.29%	***0.042***	0.73[0.54-0.99]	--	--
CR2 intron18	I	1-207653792	207653792	TG	T	20.78%	20.37%	0.53	1.03[0.94-1.12]	19.76%	20.39%	0.44	0.95[0.84-1.08]	12.18%	10.69%	0.10	1.16[0.97-1.38]	15.37%	14.53%	0.17	1.13[0.95-1.34]	0.25	1.04
CR2 intron18	I	1-207653793	207653793	GT	G	20.95%	20.64%	0.58	1.02[0.94-1.12]	19.42%	20.22%	0.36	0.94[0.83-1.07]	12.18%	10.69%	0.10	1.16[0.97-1.38]	15.37%	14.53%	0.17	1.13[0.95-1.34]	0.31	1.03
CR2 intron18	G	rs12117916	207653814	C	T	20.18%	19.85%	0.63	1.02[0.94-1.11]	20.10%	20.17%	0.79	0.98[0.87-1.11]	11.71%	10.42%	0.13	1.15[0.96-1.37]	14.94%	14.73%	0.44	1.07[0.90-1.28]	0.32	1.03
CR2 intron18	I	1-207654265	207654265	C	CA	19.67%	19.25%	0.58	1.03[0.94-1.12]	--	--	--	--	12.06%	10.48%	0.078	1.17[0.98-1.40]	13.82%	13.13%	0.22	1.12[0.93-1.35]	--	--
CR2 intron18	I	rs12059417	207654301	T	A	8.26%	9.37%	0.054	0.89[0.79-1.00]	--	--	--	--	--	--	--	--	3.80%	5.19%	0.055	0.74[0.55-1.01]	--	--
CR2 intron18	I	rs17186771	207655193	G	C	22.70%	22.11%	0.46	1.03[0.95-1.12]	6.58%	7.15%	0.25	0.89[0.73-1.09]	11.96%	10.41%	0.084	1.17[0.98-1.39]	14.30%	13.13%	0.084	1.18[0.98-1.42]	0.15	1.05
CR2 intron18	I	rs11117884	207655472	G	A	41.08%	41.13%	0.94	1.00[0.93-1.08]	--	--	--	--	--	--	--	--	--	--	--	--	--	--
CR2 intron18	I	rs1041250	207655815	A	G	22.71%	22.08%	0.42	1.04[0.95-1.12]	6.64%	7.06%	0.41	0.92[0.76-1.12]	12.13%	10.54%	0.079	1.17[0.98-1.40]	15.29%	14.20%	0.079	1.17[0.98-1.39]	0.096	1.06
CR2 intron18	G	rs6690215	207656050	C	T	42.69%	43.56%	0.28	0.96[0.90-1.03]	57.46%	58.74%	0.39	0.96[0.87-1.06]	56.36%	57.69%	0.32	0.95[0.85-1.06]	34.23%	36.78%	0.41	0.95[0.83-1.08]	0.062	0.96
CR2 intron18	G	rs7543913	207656502	T	C	22.99%	22.15%	0.25	1.05[0.97-1.14]	7.08%	7.37%	0.61	0.95[0.79-1.15]	12.40%	10.61%	***0.046***	1.19[1.00-1.42]	15.37%	14.62%	0.14	1.14[0.96-1.35]	***0.044***	1.07
CR2 intron18	G	rs6665881	207656605	C	G	40.86%	41.00%	0.95	1.00[0.93-1.07]	36.34%	34.69%	0.30	1.06[0.95-1.17]	31.16%	31.62%	0.75	0.98[0.87-1.10]	54.38%	52.53%	0.98	1.00[0.88-1.14]	0.74	1.01
CR2 intron18	I	rs61821133	207657173	A	C	7.22%	7.99%	0.15	0.91[0.80-1.03]	--	--	--	--	--	--	--	--	3.97%	5.01%	0.13	0.79[0.58-1.07]	--	--
CR2 intron18	G	rs17045468	207657375	T	C	0.78%	0.93%	0.24	0.80[0.55-1.16]	2.57%	1.72%	***0.018***	1.50[1.07-2.11]	29.31%	27.81%	0.28	1.07[0.95-1.21]	0.61%	0.75%	0.66	0.85[0.4-1.78]	0.19	1.07
CR2 intron18	G	rs12021671	207658145	A	G	35.88%	36.56%	0.39	0.97[0.90-1.04]	56.63%	57.79%	0.52	0.97[0.88-1.07]	56.36%	57.61%	0.36	0.95[0.85-1.06]	30.08%	32.09%	0.43	0.95[0.83-1.08]	0.12	0.96
CR2 intron18	I	rs71635141	207658395	T	C	7.15%	7.43%	0.94	0.99[0.87-1.14]	1.48%	1.39%	0.71	1.08[0.72-1.64]	--	--	--	--	2.51%	2.30%	0.30	1.24[0.82-1.87]	--	--
CR2 intron18	I	rs1977465	207658507	A	G	35.79%	36.51%	0.38	0.97[0.90-1.04]	56.35%	57.34%	0.65	0.98[0.88-1.08]	56.51%	57.92%	0.30	0.94[0.84-1.05]	29.19%	30.69%	0.68	0.97[0.84-1.12]	0.16	0.97
CR2 intron19	I	rs9429939	207659617	G	A	--	--	--	--	2.47%	2.07%	0.19	1.24[0.90-1.73]	--	--	--	--	--	--	--	--	--	--
CR2 intron19	I	1-207659913	207659913	CTG	C	19.06%	19.43%	0.42	0.96[0.88-1.05]	--	--	--	--	4.01%	4.07%	0.82	0.97[0.73-1.29]	15.52%	16.14%	0.88	0.99[0.84-1.17]	--	--

CR2 intron19	G	rs2182911	207660071	C	T	19.09%	19.50%	0.39	0.96[0.88-1.05]	13.15%	13.28%	0.82	1.02[0.88-1.17]	4.02%	4.18%	0.67	0.94[0.71-1.25]	15.22%	15.93%	0.83	0.98[0.83-1.16]	0.47	0.98
CR2 intron19	I	rs2182912	207660344	T	A	19.08%	19.45%	0.43	0.97[0.89-1.05]	5.56%	5.82%	0.65	0.95[0.77-1.18]	4.01%	4.11%	0.77	0.96[0.72-1.27]	15.15%	15.76%	0.90	0.99[0.84-1.17]	0.36	0.97
CR2 intron19	G	rs2182913	207660378	A	G	31.36%	32.59%	0.16	0.95[0.88-1.02]	51.79%	51.50%	0.88	1.01[0.92-1.11]	4.06%	4.35%	0.54	0.92[0.69-1.21]	24.75%	27.19%	0.21	0.91[0.79-1.05]	0.13	0.96
CR2 intron19	G	rs7519408	207661289	G	C	12.20%	12.06%	0.98	1.00[0.90-1.11]	12.33%	12.69%	0.75	1.02[0.88-1.19]	4.03%	4.15%	0.73	0.95[0.71-1.27]	11.58%	12.07%	0.69	0.96[0.79-1.17]	0.92	1.00
CR2 intron19	I	rs115749816	207661321	A	G	--	--	--	--	4.73%	5.00%	0.78	1.03[0.82-1.30]	--	--	--	--	--	--	--	--	--	--
CR2 intron19	I	rs61821134	207661446	G	A	7.49%	8.24%	0.18	0.92[0.81-1.04]	--	--	--	--	--	--	--	--	3.32%	4.42%	0.10	0.76[0.55-1.06]	--	--
CR2 intron19	I	rs76484450	207661485	G	A	5.15%	5.44%	0.86	0.99[0.85-1.15]	6.64%	6.36%	0.73	1.04[0.85-1.27]	--	--	--	--	3.70%	4.19%	0.78	0.96[0.70-1.31]	--	--
CR2 intron19	I	rs79578547	207661637	G	A	--	--	--	--	4.25%	3.53%	0.21	1.18[0.91-1.51]	--	--	--	--	--	--	--	--	--	--
CR2 intron19	G	rs4618970	207661640	C	T	12.28%	12.31%	0.76	0.98[0.89-1.09]	12.22%	12.64%	0.86	1.01[0.88-1.17]	4.01%	4.14%	0.72	0.95[0.71-1.26]	11.30%	12.00%	0.55	0.94[0.78-1.14]	0.65	0.98
CR2 intron19	I	rs61821135	207661679	C	T	7.49%	8.24%	0.18	0.92[0.81-1.04]	--	--	--	--	--	--	--	--	3.32%	4.42%	0.10	0.76[0.55-1.06]	--	--
CR2 intron19	I	rs147742969	207661861	T	C	4.39%	4.67%	0.73	0.97[0.83-1.14]	6.64%	6.36%	0.73	1.04[0.85-1.27]	--	--	--	--	3.70%	4.19%	0.78	0.96[0.70-1.31]	--	--
CR2 intron19	I	rs12068225	207662306	A	G	--	--	--	--	5.84%	5.74%	0.75	1.04[0.84-1.28]	--	--	--	--	--	--	--	--	--	--
CR2 3'UTR	I	rs9429940	207662912	T	C	12.24%	12.28%	0.77	0.98[0.89-1.09]	12.22%	12.62%	0.84	1.02[0.88-1.17]	4.01%	4.11%	0.77	0.96[0.72-1.27]	11.24%	11.74%	0.68	0.96[0.79-1.16]	0.73	0.99
CR1 5' upstream	G	rs17045761	207663356	A	G	5.78%	5.13%	0.19	1.11[0.95-1.29]	4.71%	5.29%	0.24	0.88[0.70-1.09]	17.41%	19.30%	0.10	0.89[0.77-1.02]	6.58%	5.59%	0.089	1.25[0.97-1.63]	0.88	0.99
CR1 5' upstream	I	rs4525038	207663937	T	C	12.32%	12.36%	0.74	0.98[0.89-1.09]	--	--	--	--	4.01%	4.11%	0.77	0.96[0.72-1.27]	12.18%	12.69%	0.61	0.95[0.79-1.15]	--	--
CR1 5' upstream	I	rs6663887	207664423	A	G	24.55%	25.41%	0.32	0.96[0.89-1.04]	--	--	--	--	4.02%	4.20%	0.67	0.94[0.71-1.25]	20.64%	22.94%	0.13	0.89[0.77-1.04]	--	--
CR1 5' upstream	I	rs6700109	207664607	C	T	16.67%	16.99%	0.63	0.98[0.89-1.07]	--	--	--	--	4.01%	4.11%	0.77	0.96[0.72-1.27]	15.53%	16.56%	0.55	0.95[0.80-1.12]	--	--
CR1 5' upstream	I	rs9429779	207664721	G	A	31.34%	32.61%	0.14	0.95[0.88-1.02]	51.77%	51.62%	0.96	1.00[0.91-1.10]	4.02%	4.20%	0.67	0.94[0.71-1.25]	24.52%	27.03%	0.19	0.91[0.79-1.05]	0.11	0.96
CR1 5' upstream	I	rs61821136	207665084	G	C	7.47%	8.24%	0.16	0.91[0.81-1.04]	--	--	--	--	--	--	--	--	3.32%	4.42%	0.10	0.76[0.55-1.05]	--	--
CR1 5' upstream	I	rs115883226	207665226	G	A	--	--	--	--	4.73%	5.00%	0.78	1.03[0.82-1.30]	--	--	--	--	--	--	--	--	--	--
CR1 5' upstream	I	rs10863336	207665920	T	C	31.07%	30.86%	0.92	1.00[0.92-1.07]	--	--	--	--	--	--	--	--	--	--	--	--	--	--
CR1 5' upstream	G	rs4618971	207666061	C	T	23.69%	24.21%	0.47	0.97[0.90-1.05]	27.79%	27.70%	0.65	1.03[0.92-1.14]	4.01%	4.18%	0.66	0.94[0.71-1.25]	19.93%	21.27%	0.62	0.96[0.83-1.12]	0.58	0.98
CR1 5' upstream	I	rs1410408	207666308	A	G	6.19%	5.73%	0.52	1.05[0.91-1.21]	7.30%	7.65%	0.57	0.95[0.79-1.14]	--	--	--	--	6.51%	5.61%	0.11	1.24[0.96-1.61]	--	--
CR1 5' upstream	I	rs11807805	207666597	G	C	4.16%	4.43%	0.80	0.98[0.83-1.16]	--	--	--	--	--	--	--	--	3.64%	4.07%	0.81	0.96[0.70-1.33]	--	--
CR1 5' upstream	I	rs9429780	207667190	G	C	23.32%	24.08%	0.31	0.96[0.89-1.04]	--	--	--	--	4.01%	4.11%	0.77	0.96[0.72-1.27]	19.63%	20.70%	0.77	0.98[0.84-1.14]	--	--
CR1 5' upstream	I	rs9429781	207667222	G	T	23.32%	24.08%	0.31	0.96[0.89-1.04]	--	--	--	--	4.01%	4.11%	0.77	0.96[0.72-1.27]	19.15%	20.39%	0.70	0.97[0.83-1.13]	--	--
CR1 5' upstream	I	rs9429941	207667303	T	A	23.33%	24.08%	0.32	0.96[0.89-1.04]	--	--	--	--	4.01%	4.11%	0.77	0.96[0.72-1.27]	19.63%	20.62%	0.82	0.98[0.84-1.15]	--	--
CR1 5' upstream	I	rs115257007	207667495	A	C	3.15%	3.53%	0.72	0.97[0.80-1.17]	--	--	--	--	--	--	--	--	3.64%	4.02%	0.88	0.97[0.71-1.35]	--	--
CR1 5' upstream	I	rs12078329	207667497	T	C	4.24%	4.52%	0.76	0.97[0.82-1.15]	--	--	--	--	--	--	--	--	4.75%	4.80%	0.84	1.03[0.77-1.39]	--	--
CR1 5' upstream	I	1-207667501	207667501	G	GA	3.40%	3.74%	0.73	0.97[0.80-1.17]	--	--	--	--	--	--	--	--	--	--	--	--	--	--
CR1 5' upstream	I	rs76124527	207667549	G	A	4.24%	4.52%	0.76	0.97[0.82-1.15]	14.07%	13.74%	0.63	1.04[0.90-1.19]	--	--	--	--	4.42%	4.49%	0.78	1.04[0.77-1.41]	--	--
CR1 5' upstream	I	rs11117911	207667985	A	G	4.27%	4.52%	0.75	0.97[0.82-1.15]	--	--	--	--	--	--	--	--	4.75%	4.80%	0.84	1.03[0.77-1.39]	--	--
CR1 5' upstream	I	rs11117913	207668235	T	C	4.24%	4.52%	0.76	0.97[0.82-1.15]	--	--	--	--	--	--	--	--	4.75%	4.79%	0.83	1.03[0.77-1.39]	--	--
CR1 5' upstream	I	rs80141998	207668315	A	G	4.14%	4.43%	0.73	0.97[0.82-1.15]	6.42%	5.92%	0.48	1.08[0.88-1.32]	--	--	--	--	3.64%	4.02%	0.88	0.98[0.71-1.35]	--	--
CR1 5' upstream	I	rs7525160	207668414	C	G	31.59%	31.46%	0.94	1.00[0.93-1.07]	--	--	--	--	--	--	--	--	--	--	--	--	--	--
CR1 5' upstream	I	rs7525170	207668447	G	A	19.06%	19.51%	0.39	0.96[0.88-1.05]	--	--	--	--	4.01%	4.11%	0.77	0.96[0.72-1.27]	14.75%	15.60%	0.77	0.98[0.82-1.16]	--	--
CR1 5' upstream	I	1-207668549	207668549	T	TTTTC	4.14%	4.43%	0.73	0.97[0.82-1.15]	6.42%	6.01%	0.58	1.06[0.86-1.30]	--	--	--	--	3.64%	4.02%	0.88	0.98[0.71-1.35]	--	--
CR1 5' upstream	I	rs9429942	207668630	C	T	--	--	--	--	51.51%	51.21%	0.90	1.01[0.91-1.11]	4.01%	4.30%	0.54	0.92[0.69-1.22]	24.10%	26.70%	0.19	0.91[0.79-1.05]	--	--
CR1 5' upstream	I	rs55819427	207669111	T	C	7.45%	8.18%	0.19	0.92[0.81-1.04]	--	--	--	--	--	--	--	--	4.56%	5.57%	0.14	0.81[0.61-1.07]	--	--
CR1 5' upstream	I	rs61522287	207669248	A	G	4.14%	4.43%	0.73	0.97[0.82-1.15]	6.42%	5.98%	0.55	1.06[0.87-1.30]	--	--	--	--	3.64%	4.02%	0.88	0.98[0.71-1.35]	--	--
CR1 5' upstream	I	rs61821138	207669343	A	G	11.98%	12.87%	0.28	0.94[0.85-1.05]	--	--	--	--	--	--	--	--	9.22%	10.82%	0.15	0.86[0.70-1.06]	--	--
CR1 intron1	I	rs9429943	207669924	C	G	24.22%	25.14%	0.30	0.96[0.89-1.04]	50.51%	50.52%	0.99	1.00[0.91-1.10]	4.02%	4.23%	0.62	0.93[0.70-1.24]	20.08%	22.34%	0.13	0.89[0.76-1.04]	0.17	0.96
CR1 intron1	I	rs115050353	207670281	C	A	4.14%	4.42%	0.75	0.97[0.82-1.15]	6.42%	6.01%	0.58	1.06[0.86-1.30]	--	--	--	--	3.63%	4.00%	0.88	0.98[0.71-1.35]	--	--
CR1 intron1	I	rs111449973	207670307	T	A	24.22%	25.13%	0.30	0.96[0.89-1.04]	50.42%	50.52%	0.94	1.00[0.90-1.10]	4.02%	4.23%	0.62	0.93[0.70-1.24]	20.08%	22.34%	0.13	0.89[0.76-1.04]	0.16	0.96
CR1 intron1	G	rs112822892	207670438	G	A	30.79%	30.24%	0.31	1.04[0.96-1.12]	8.93%	9.44%	0.55	0.95[0.80-1.13]	12.09%	10.68%	0.14	1.14[0.96-1.36]	18.79%	17.73%	***0.047***	1.18[1.00-1.38]	0.067	1.06
CR1 intron1	G	rs113977956	207670744	C	T	12.03%	12.88%	0.30	0.95[0.85-1.05]	38.19%	37.91%	0.86	0.99[0.90-1.09]	0.04%	0.16%	0.27	0.29[0.03-2.61]	9.40%	11.03%	0.14	0.86[0.70-1.05]	0.19	0.96
CR1 intron1	G	rs113151130	207670928	G	A	11.51%	12.58%	0.16	0.93[0.84-1.03]	38.09%	37.55%	0.98	1.00[0.91-1.11]	0.04%	0.16%	0.27	0.29[0.03-2.61]	9.06%	10.46%	0.24	0.88[0.72-1.09]	0.19	0.96
CR1 intron1	G	rs112585529	207671269	C	T	18.81%	19.37%	0.32	0.96[0.88-1.04]	2.60%	2.52%	0.88	0.98[0.72-1.33]	4.02%	4.14%	0.72	0.95[0.72-1.26]	13.75%	14.93%	0.54	0.95[0.80-1.13]	0.22	0.96
CR1 intron1	G	rs113901708	207671476	C	T	18.69%	19.12%	0.35	0.96[0.88-1.05]	8.36%	8.42%	0.88	0.99[0.83-1.18]	4.01%	4.11%	0.76	0.96[0.72-1.27]	14.13%	15.13%	0.59	0.95[0.80-1.13]	0.28	0.96
CR1 intron1	I	rs115808230	207671621	A	G	7.44%	8.14%	0.21	0.92[0.81-1.05]	--	--	--	--	--	--	--	--	3.31%	4.43%	0.10	0.76[0.55-1.05]	--	--
CR1 intron1	G	rs112981697	207671849	A	C	12.01%	12.84%	0.28	0.94[0.85-1.05]	38.33%	38.04%	0.90	0.99[0.90-1.10]	--	--	--	--	9.38%	10.91%	0.16	0.87[0.70-1.06]	--	--
CR1 intron1	I	rs114715994	207672533	T	C	--	--	--	--	3.14%	3.84%	***0.040***	0.75[0.58-0.99]	--	--	--	--	--	--	--	--	--	--
CR1 intron1	I	rs113379492	207672612	G	A	24.23%	25.13%	0.31	0.96[0.89-1.04]	50.39%	50.55%	0.91	0.99[0.90-1.10]	4.02%	4.19%	0.66	0.94[0.71-1.25]	19.91%	22.39%	0.089	0.88[0.75-1.02]	0.14	0.96
CR1 intron1	I	rs61822962	207672719	T	G	11.99%	12.84%	0.30	0.95[0.85-1.05]	38.10%	38.00%	0.74	0.98[0.89-1.09]	--	--	--	--	9.28%	10.88%	0.15	0.86[0.70-1.06]	--	--
CR1 intron1	I	rs59179545	207672988	C	G	11.98%	12.84%	0.29	0.95[0.85-1.05]	38.10%	38.00%	0.74	0.98[0.89-1.09]	--	--	--	--	9.28%	10.88%	0.15	0.86[0.70-1.06]	--	--
CR1 intron1	I	rs112381793	207673084	A	T	24.22%	25.13%	0.30	0.96[0.89-1.04]	50.39%	50.60%	0.86	0.99[0.90-1.09]	4.02%	4.16%	0.72	0.95[0.71-1.26]	19.89%	22.39%	0.086	0.88[0.75-1.02]	0.13	0.96
CR1 intron1	I	rs61822963	207673414	A	G	7.42%	8.14%	0.20	0.92[0.81-1.04]	--	--	--	--	--	--	--	--	3.73%	4.83%	0.11	0.78[0.57-1.06]	--	--
CR1 intron1	I	rs112049645	207673631	T	C	24.33%	25.24%	0.32	0.96[0.89-1.04]	50.24%	50.50%	0.82	0.99[0.90-1.09]	4.02%	4.16%	0.72	0.95[0.71-1.26]	19.87%	22.39%	0.083	0.87[0.75-1.02]	0.12	0.96
CR1 intron1	I	rs113012055	207673945	A	G	18.82%	19.28%	0.39	0.96[0.88-1.05]	2.43%	2.42%	0.75	0.95[0.69-1.30]	4.01%	4.07%	0.82	0.97[0.73-1.29]	14.18%	15.42%	0.51	0.94[0.80-1.12]	0.26	0.96
CR1 intron1	G	rs113703567	207673980	A	G	12.04%	12.92%	0.27	0.94[0.85-1.05]	37.99%	38.04%	0.68	0.98[0.89-1.08]	0.04%	0.16%	0.27	0.29[0.03-2.61]	9.45%	11.01%	0.15	0.86[0.70-1.06]	0.13	0.95
CR1 intron1	I	rs114467562	207674503	T	C	--	--	--	--	1.23%	1.36%	0.85	0.96[0.62-1.48]	--	--	--	--	--	--	--	--	--	--
CR1 intron1	G	rs112741151	207675655	A	G	31.21%	30.45%	0.21	1.05[0.97-1.13]	23.77%	24.20%	0.84	0.99[0.88-1.11]	12.57%	11.13%	0.14	1.14[0.96-1.35]	20.26%	19.14%	***0.045***	1.17[1.00-1.37]	***0.043***	1.06
CR1 intron1	I	rs61822964	207675712	G	A	7.55%	8.09%	0.36	0.94[0.83-1.07]	--	--	--	--	--	--	--	--	--	--	--	--	--	--
CR1 intron1	I	rs111445330	207675853	G	T	11.91%	12.74%	0.30	0.95[0.85-1.05]	37.98%	37.82%	0.81	0.99[0.90-1.09]	--	--	--	--	9.04%	10.75%	0.12	0.85[0.69-1.04]	--	--
CR1 intron1	I	rs61822965	207676203	G	A	6.59%	7.10%	0.25	0.92[0.81-1.06]	--	--	--	--	--	--	--	--	--	--	--	--	--	--

Abbreviation: Freq, allele frequency; OR, odds ratio; CI, confidence interval; ND, not determined; I, imputed; G, genotyped; CTRL, control; EA, European American; AA, African American; AS, Asian; HS, Hispanic; Meta, Meta-analysis
Position is based on GRCh37/hg19.

Table S2. Association of *CR2* variants with dsDNA autoantibodies in multiple ancestral groups

						EA							AA							HS						Meta	
						Freq							Freq							Freq							
Annotation	Type	SNP	NCBI 37	A1	A2	Anti-dsDNA+	Ctrl	P	OR	L95	U95	P_c	Anti-dsDNA+	Ctrl	P	OR	L95	U95	P_c	Anti-dsDNA+	Ctrl	P	OR	L95	U95	*P*	OR
CR2 5' upstream	I	rs311321	207618591	A	G	27.0%	30.6%	5.9E-03	0.86	0.77	0.96	0.24	--	--	--	--	--	--		21.3%	21.2%	0.4661	1.07	0.89	1.28	--	--
CR2 5' upstream	I	rs311320	207618661	G	T	27.2%	30.6%	9.2E-03	0.87	0.78	0.97	0.31	41.3%	43.0%	0.20	0.92	0.81	1.05		21.6%	21.5%	0.5016	1.06	0.89	1.27	0.104	0.94
CR2 5' upstream	I	rs73089144	207618967	G	A	--	--	--	--	--	--		9.2%	8.8%	0.44	1.09	0.87	1.37		--	--	--	--	--	--	--	--
CR2 5' upstream	I	rs59808407	207619051	T	C	--	--	--	--	--	--		9.2%	8.8%	0.44	1.09	0.87	1.37		--	--	--	--	--	--	--	--
CR2 5' upstream	I	rs138684626	207619203	G	A	--	--	--	--	--	--		2.4%	2.8%	0.51	0.87	0.58	1.31		--	--	--	--	--	--	--	--
CR2 5' upstream	I	rs57767408	207619399	C	T	--	--	--	--	--	--		3.3%	2.7%	0.36	1.19	0.82	1.72		--	--	--	--	--	--	--	--
CR2 5' upstream	I	rs17044032	207619949	G	A	7.1%	9.9%	6.6E-04	0.73	0.61	0.88	ND	10.6%	10.7%	0.52	0.93	0.76	1.15		4.3%	5.6%	0.1516	0.78	0.55	1.10	--	--
CR2 5' upstream	I	rs114928704	207620136	A	C	--	--	--	--	--	--		5.1%	4.2%	0.25	1.19	0.88	1.60		--	--	--	--	--	--	--	--
CR2 5' upstream	I	rs12081383	207620289	C	A	--	--	--	--	--	--		9.2%	8.8%	0.44	1.09	0.87	1.37		--	--	--	--	--	--	--	--
CR2 5' upstream	I	rs311319	207620401	G	A	26.9%	30.4%	6.2E-03	0.86	0.77	0.96	0.23	40.6%	42.2%	0.24	0.93	0.81	1.05		21.3%	21.2%	0.4661	1.07	0.89	1.28	0.1014	0.94
CR2 5' upstream	I	rs148632661	207620900	G	T	--	--	--	--	--	--		2.4%	2.8%	0.51	0.87	0.58	1.31		--	--	--	--	--	--	--	--
CR2 5' upstream	I	rs184612141	207621755	G	A	--	--	--	--	--	--		1.6%	1.5%	0.79	1.07	0.63	1.81		--	--	--	--	--	--	--	--
CR2 5' upstream	I	rs311317	207621832	A	G	27.1%	30.5%	9.1E-03	0.87	0.78	0.97	0.29	51.4%	52.7%	0.42	0.95	0.84	1.08		22.4%	22.1%	0.4647	1.07	0.89	1.28	0.1792	0.95
CR2 5' upstream	I	rs311316	207622223	A	T	11.6%	11.5%	0.93	0.99	0.85	1.16		--	--	--	--	--	--		9.1%	10.2%	0.3471	0.89	0.69	1.14	--	--
CR2 5' upstream	I	rs311315	207622318	G	T	11.6%	11.5%	0.93	0.99	0.85	1.16		--	--	--	--	--	--		9.1%	10.2%	0.3471	0.89	0.69	1.14	--	--
CR2 5' upstream	I	rs191006951	207622587	T	C	--	--	--	--	--	--		1.5%	1.4%	0.78	1.08	0.63	1.85		--	--	--	--	--	--	--	--
CR2 5' upstream	I	rs17044219	207623781	A	G	1.3%	1.1%	0.43	1.19	0.77	1.86		--	--	--	--	--	--		--	--	--	--	--	--	--	--
CR2 5' upstream	I	rs7527218	207623829	G	A	44.7%	46.7%	0.13	0.93	0.84	1.02		71.4%	73.2%	0.33	0.93	0.80	1.08		--	--	--	--	--	--	--	--
CR2 5' upstream	I	rs311314	207623868	G	A	11.6%	11.5%	0.93	0.99	0.85	1.16		--	--	--	--	--	--		9.1%	10.2%	0.3471	0.89	0.69	1.14	--	--
CR2 5' upstream	I	rs61240730	207623930	C	T	7.1%	9.9%	6.6E-04	0.73	0.61	0.88	ND	5.7%	6.4%	0.13	0.81	0.61	1.07		4.3%	5.5%	0.1982	0.80	0.56	1.13	--	--
CR2 5' upstream	I	rs71635139	207624289	A	T	6.9%	7.2%	0.93	0.99	0.82	1.20		--	--	--	--	--	--		--	--	--	--	--	--	--	--
CR2 5' upstream	I	rs72644189	207624395	T	C	--	--	--	--	--	--		--	--	--	--	--	--		--	--	--	--	--	--	--	--
CR2 5' upstream	I	rs11808746	207624861	T	A	--	--	--	--	--	--		2.3%	1.7%	0.18	1.36	0.87	2.13		1.0%	1.3%	0.9804	1.01	0.51	2.01	--	--
CR2 5' upstream	I	rs311313	207624911	C	T	26.9%	30.4%	6.2E-03	0.86	0.77	0.96	0.23	40.3%	42.1%	0.20	0.92	0.81	1.05		21.3%	21.2%	0.4661	1.07	0.89	1.28	0.08911	0.94
CR2 5' upstream	I	rs188721882	207625219	C	T	--	--	--	--	--	--		1.5%	1.4%	0.78	1.08	0.63	1.85		--	--	--	--	--	--	--	--
CR2 5' upstream	I	1-207625357	207625357	TAA	T	--	--	--	--	--	--		--	--	--	--	--	--		5.9%	5.3%	0.41	1.14	0.83	1.58	--	--
CR2 5' upstream	I	rs143193885	207625498	G	A	--	--	--	--	--	--		1.6%	1.5%	0.79	1.07	0.63	1.81		--	--	--	--	--	--	--	--
CR2 5' upstream	I	rs311312	207625541	G	T	11.6%	11.5%	0.93	0.99	0.85	1.16		--	--	--	--	--	--		9.1%	10.2%	0.3471	0.89	0.69	1.14	--	--
CR2 5' upstream	I	rs144800606	207625690	C	T	--	--	--	--	--	--		1.5%	1.4%	0.78	1.08	0.63	1.85		--	--	--	--	--	--	--	--
CR2 5' upstream	I	rs151025105	207626329	A	T	--	--	--	--	--	--		1.5%	1.4%	0.78	1.08	0.63	1.85		--	--	--	--	--	--	--	--
CR2 5' upstream	I	rs140024463	207626613	A	C	--	--	--	--	--	--		1.6%	1.5%	0.79	1.07	0.63	1.81		--	--	--	--	--	--	--	--
CR2 5' upstream	I	rs143641831	207626646	A	G	--	--	--	--	--	--		1.5%	1.4%	0.78	1.08	0.63	1.85		--	--	--	--	--	--	--	--
CR2 5' upstream	G	rs12135588	207627057	G	A	2.2%	2.9%	0.10	0.76	0.55	1.06		0.8%	0.3%	0.020	2.85	1.18	6.90	ND	1.2%	1.2%	0.9127	1.04	0.53	2.03	--	--
CR2 5' upstream	I	rs140630338	207627293	G	T	--	--	--	--	--	--		1.6%	1.5%	0.79	1.07	0.63	1.81		--	--	--	--	--	--	--	--
CR2 5'UTR	I	rs182309299	207627672	T	C	--	--	--	--	--	--		2.4%	2.8%	0.51	0.87	0.58	1.31		--	--	--	--	--	--	--	--
CR2 5'UTR	G	rs3813946	207627693	C	T	18.3%	19.2%	0.43	0.95	0.84	1.08		6.9%	8.2%	0.10	0.81	0.63	1.04		13.8%	12.8%	0.1385	1.18	0.95	1.46	0.7946	1.01
CR2 intron1	G	rs1876453	207627918	A	G	7.1%	9.9%	6.6E-04	0.73	0.61	0.88	--	6.7%	8.6%	9.7E-03	0.72	0.56	0.92	--	3.0%	5.1%	7.1E-03	0.58	0.39	0.86	0.00000076	0.71
CR2 intron1	G	rs17258955	207627998	T	C	3.7%	4.0%	0.33	0.88	0.68	1.14		1.0%	0.5%	0.056	2.03	0.98	4.17		1.7%	1.8%	0.9692	0.99	0.57	1.73	--	--
CR2 intron1	G	rs41304115	207628076	A	G	3.1%	3.3%	0.65	0.94	0.71	1.24		--	--	--	--	--	--		1.2%	1.4%	0.917	0.97	0.51	1.82	--	--
CR2 intron1	G	rs41304111	207628347	G	A	2.2%	2.2%	0.99	1.00	0.72	1.39		0.3%	0.4%	0.78	0.85	0.27	2.70		1.9%	1.1%	0.06806	1.77	0.96	3.26	--	--
CR2 intron1	I	rs116762805	207628788	T	A	--	--	--	--	--	--		5.1%	4.2%	0.25	1.19	0.88	1.60		--	--	--	--	--	--	--	--
CR2 intron1	G	rs311302	207629416	T	C	27.4%	30.7%	9.9E-03	0.87	0.78	0.97	0.36	42.0%	44.0%	0.16	0.91	0.80	1.04		21.9%	21.8%	0.491	1.06	0.89	1.27	0.07855	0.94
CR2 intron1	G	rs311303	207629514	G	A	27.6%	30.7%	0.019	0.88	0.79	0.98	0.51	51.4%	52.7%	0.44	0.95	0.84	1.08		22.8%	22.7%	0.4931	1.06	0.89	1.27	0.2238	0.96
CR2 intron1	I	rs311304	207629702	A	C	27.0%	30.5%	7.6E-03	0.86	0.78	0.96	0.27	40.3%	42.1%	0.20	0.92	0.81	1.05		21.4%	21.3%	0.4754	1.07	0.89	1.28	0.09598	0.94
CR2 intron1	I	rs146939930	207629863	G	A	--	--	--	--	--	--		1.6%	1.5%	0.79	1.07	0.63	1.81		--	--	--	--	--	--	--	--
CR2 intron1	G	rs311305	207630032	G	A	0.0%	0.1%	1.00	0.00	0.00	inf		11.3%	11.2%	0.85	1.02	0.83	1.25		0.7%	0.7%	0.8141	0.90	0.38	2.16	--	--
CR2 intron1	G	rs311306	207630444	C	G	26.9%	30.4%	7.2E-03	0.86	0.78	0.96	0.28	40.2%	42.1%	0.20	0.92	0.81	1.05		21.5%	21.4%	0.4622	1.07	0.89	1.28	0.09485	0.94
CR2 intron1	I	rs150868219	207630836	T	C	--	--	--	--	--	--		1.6%	1.5%	0.79	1.07	0.63	1.81		--	--	--	--	--	--	--	--
CR2 intron1	G	rs61821130	207630888	A	C	7.1%	9.9%	8.0E-04	0.73	0.61	0.88	ND	11.5%	12.2%	0.24	0.89	0.73	1.08		4.8%	6.0%	0.1956	0.80	0.58	1.12	0.0004259	0.80
CR2 intron1	I	rs73089153	207631048	A	T	--	--	--	--	--	--		9.5%	8.9%	0.35	1.11	0.89	1.39		--	--	--	--	--	--	--	--
CR2 intron1	G	rs12140602	207631158	A	T	19.9%	20.5%	0.62	0.97	0.86	1.09		8.1%	9.4%	0.14	0.84	0.67	1.06		14.9%	14.0%	0.1424	1.17	0.95	1.43	0.7349	1.01
CR2 intron1	G	rs12139795	207631205	G	A	19.8%	20.4%	0.58	0.97	0.86	1.09		15.0%	15.7%	0.58	0.95	0.79	1.14		15.6%	14.1%	0.06549	1.21	0.99	1.48	0.4762	1.03
CR2 intron1	I	rs80182520	207631332	T	A	--	--	--	--	--	--		6.8%	6.4%	0.44	1.11	0.86	1.43		--	--	--	--	--	--	--	--
CR2 intron1	I	rs146796037	207631753	T	C	--	--	--	--	--	--		1.6%	1.5%	0.80	1.07	0.63	1.81		--	--	--	--	--	--	--	--
CR2 intron1	G	rs311307	207631778	T	C	27.0%	30.6%	6.8E-03	0.86	0.78	0.96	0.29	39.9%	42.1%	0.14	0.91	0.80	1.03		21.5%	21.5%	0.5129	1.06	0.89	1.27	0.07053	0.94
CR2 intron1	I	rs77276388	207631951	C	T	--	--	--	--	--	--		3.9%	3.4%	0.49	1.13	0.80	1.58		--	--	--	--	--	--	--	--
CR2 intron1	I	rs77625468	207632129	C	G	--	--	--	--	--	--		2.5%	2.3%	0.62	1.11	0.73	1.68		--	--	--	--	--	--	--	--
CR2 intron1	I	rs311308	207632487	A	G	--	--	--	--	--	--		11.3%	11.3%	0.86	1.02	0.83	1.25		--	--	--	--	--	--	--	--
CR2 intron1	I	rs142880184	207632526	C	G	--	--	--	--	--	--		1.5%	1.4%	0.77	1.08	0.63	1.85		--	--	--	--	--	--	--	--
CR2 intron1	G	rs17044525	207632796	G	A	7.4%	9.9%	2.8E-03	0.76	0.64	0.91	ND	13.3%	13.6%	0.37	0.92	0.76	1.11		5.0%	6.1%	0.2618	0.83	0.60	1.15	0.002683	0.83
CR2 intron1	I	rs150973349	207632805	C	T	--	--	--	--	--	--		1.5%	1.4%	0.77	1.08	0.63	1.85		--	--	--	--	--	--	--	--
CR2 intron1	I	rs116542838	207633194	C	T	--	--	--	--	--	--		5.1%	4.2%	0.25	1.19	0.88	1.60		--	--	--	--	--	--	--	--
CR2 intron1	I	rs146923426	207633875	G	A	--	--	--	--	--	--		1.5%	1.4%	0.77	1.08	0.63	1.85		--	--	--	--	--	--	--	--
CR2 intron1	I	rs149048095	207634274	C	T	--	--	--	--	--	--		1.5%	1.4%	0.77	1.08	0.63	1.85		--	--	--	--	--	--	--	--
CR2 intron1	I	rs143079233	207634294	G	A	--	--	--	--	--	--		1.5%	1.4%	0.77	1.08	0.63	1.85		--	--	--	--	--	--	--	--
CR2 intron1	I	1-207634463	207634463	G	GT	--	--	--	--	--	--		3.9%	3.4%	0.49	1.13	0.80	1.58		--	--	--	--	--	--	--	--
CR2 intron1	I	rs141941832	207634483	T	G	--	--	--	--	--	--		1.5%	1.4%	0.71	1.11	0.65	1.90		--	--	--	--	--	--	--	--
CR2 intron1	I	rs74857976	207634617	G	A	--	--	--	--	--	--		5.1%	4.2%	0.25	1.19	0.88	1.60		--	--	--	--	--	--	--	--
CR2 intron1	G	rs17044576	207634670	C	T	5.8%	4.4%	0.031	1.27	1.02	1.58	0.06	12.2%	11.5%	0.24	1.13	0.92	1.37		5.7%	5.2%	0.5423	1.11	0.80	1.53	0.3517	1.05
CR2 intron1	G	rs311309	207635087	G	A	0.0%	0.1%	1.00	0.00	0.00	inf		11.4%	11.3%	0.79	1.03	0.84	1.26		0.8%	0.8%	0.8309	0.91	0.40	2.11	--	--

CR2 intron1	I	rs146134620	207635390	A	G	--	--	--	--	--	--		1.5%	1.4%	0.71	1.11	0.65	1.90	--	--	--	--	--	--	--	--
CR2 intron1	G	rs311310	207635573	A	G	26.6%	30.4%	3.7E-03	0.85	0.76	0.95	0.18	37.9%	39.4%	0.25	0.93	0.82	1.06	21.0%	20.7%	0.3549	1.09	0.91	1.31	0.1049	0.94
CR2 intron1	G	rs1567190	207635789	T	C	47.5%	49.5%	0.099	0.92	0.84	1.02		74.3%	75.8%	0.42	0.94	0.81	1.09	39.7%	40.4%	0.7719	1.02	0.88	1.19	0.1848	0.96
CR2 intron1	I	rs3767934	207636208	T	G	19.8%	20.3%	0.59	0.97	0.86	1.09		6.9%	8.3%	0.10	0.81	0.63	1.04	14.5%	13.3%	0.09382	1.20	0.97	1.47	0.7099	1.02
CR2 intron1	G	rs3767933	207637359	T	C	47.5%	49.6%	0.069	0.91	0.83	1.01		74.5%	75.8%	0.50	0.95	0.82	1.10	39.6%	40.3%	0.7609	1.02	0.88	1.19	0.1939	0.96
CR2 intron1	I	1-207638763	207638763	T	TA	--	--	--	--	--	--		3.9%	4.0%	0.95	0.99	0.71	1.37	--	--	--	--	--	--	--	--
CR2 intron1	I	rs115281878	207639384	G	A	--	--	--	--	--	--		3.9%	4.0%	0.98	1.00	0.72	1.38	--	--	--	--	--	--	--	--
CR2 intron1	G	rs311311	207639695	C	G	27.5%	30.5%	0.018	0.88	0.79	0.98	0.48	50.3%	51.3%	0.61	0.97	0.85	1.10	22.7%	22.9%	0.6712	1.04	0.87	1.23	0.2395	0.96
CR2 intron2	I	rs145095346	207640274	G	A	--	--	--	--	--	--		3.9%	4.0%	0.99	1.00	0.72	1.39	--	--	--	--	--	--	--	--
CR2 intron2	G	rs1032980	207640345	G	T	27.4%	30.6%	0.013	0.87	0.79	0.97	0.40	42.0%	43.4%	0.31	0.94	0.82	1.06	21.8%	21.8%	0.5244	1.06	0.89	1.26	0.1475	0.95
CR2 intron2	I	rs1257562	207640492	C	T	--	--	--	--	--	--		11.3%	11.4%	0.99	1.00	0.81	1.23	--	--	--	--	--	--	--	--
CR2 intron2	I	rs1270549	207640673	A	C	27.3%	30.5%	0.013	0.87	0.78	0.97	0.38	42.0%	43.4%	0.32	0.94	0.82	1.07	21.5%	21.6%	0.541	1.06	0.89	1.26	0.1511	0.95
CR2 intron2	I	rs1032979	207641023	C	T	27.3%	30.5%	0.013	0.87	0.78	0.97	0.38	40.9%	42.4%	0.32	0.94	0.82	1.07	21.5%	21.5%	0.5211	1.06	0.89	1.27	0.1578	0.95
CR2 intron2	I	rs17045028	207641114	G	A	--	--	--	--	--	--		3.9%	4.0%	1.00	1.00	0.72	1.39	--	--	--	--	--	--	--	--
CR2 intron2	I	1-207641329	207641329	T	TTAAG	--	--	--	--	--	--		7.7%	7.2%	0.42	1.11	0.86	1.42	--	--	--	--	--	--	--	--
CR2 intron2	I	rs2063143	207641840	T	C	19.8%	20.3%	0.61	0.97	0.86	1.09		7.4%	8.5%	0.17	0.84	0.66	1.07	14.6%	13.7%	0.1342	1.17	0.95	1.44	0.69	1.02
CR2 intron3	I	rs17045039	207642125	T	A	--	--	--	--	--	--		3.8%	3.9%	0.99	1.00	0.72	1.39	--	--	--	--	--	--	--	--
CR2 intron4	G	rs1507764	207642295	A	G	19.7%	20.5%	0.53	0.96	0.85	1.09		14.2%	15.1%	0.47	0.93	0.78	1.12	15.5%	14.4%	0.1335	1.17	0.95	1.43	0.7396	1.01
CR2 intron6	I	rs11117872	207643566	C	T	--	--	--	--	--	--		6.9%	5.8%	0.25	1.17	0.90	1.51	--	--	--	--	--	--	--	--
CR2 intron6	I	rs568515	207643673	C	T	27.0%	30.5%	7.6E-03	0.86	0.78	0.96	0.27	36.7%	38.0%	0.33	0.94	0.82	1.07	20.8%	20.8%	0.4865	1.07	0.89	1.28	0.1352	0.95
CR2 intron6	G	rs10863322	207643949	G	A	45.0%	46.8%	0.16	0.93	0.85	1.03		70.4%	72.5%	0.26	0.92	0.80	1.06	38.4%	39.1%	0.8225	1.02	0.88	1.18	0.08187	0.95
CR2 intron6	I	rs17045132	207643983	A	G	--	--	--	--	--	--		3.3%	3.8%	0.59	0.91	0.64	1.29	--	--	--	--	--	--	--	--
CR2 intron9	I	rs511678	207645282	C	G	27.0%	30.5%	8.3E-03	0.87	0.78	0.96	0.28	36.7%	38.0%	0.33	0.94	0.82	1.07	20.7%	20.9%	0.5566	1.06	0.88	1.26	0.1278	0.95
CR2 intron9	I	rs116421473	207645361	A	G	--	--	--	--	--	--		6.8%	6.4%	0.44	1.11	0.86	1.43	--	--	--	--	--	--	--	--
CR2 intron9	G	rs2940253	207645743	C	G	27.4%	30.6%	0.012	0.87	0.78	0.97	0.31	36.6%	38.0%	0.28	0.93	0.82	1.06	20.8%	21.0%	0.6112	1.05	0.88	1.26	0.1438	0.95
CR2 intron9	I	rs9429774	207645895	A	G	27.0%	30.5%	7.8E-03	0.87	0.78	0.96	0.28	36.7%	38.0%	0.34	0.94	0.82	1.07	20.9%	20.9%	0.5046	1.06	0.89	1.27	0.1332	0.95
CR2 intron9	I	rs9429936	207645956	A	C	27.3%	30.5%	0.012	0.87	0.78	0.97	0.37	39.6%	41.5%	0.21	0.92	0.81	1.05	21.5%	21.5%	0.4883	1.07	0.89	1.27	0.1221	0.95
CR2 exon10	G	rs34349246	207646163	T	C	1.9%	1.4%	0.11	1.36	0.94	1.99		3.2%	3.2%	0.78	1.05	0.73	1.51	1.7%	1.6%	0.6146	1.16	0.66	2.04	0.1581	1.19
CR2 exon10	I	rs1048971	207646322	A	G	32.8%	34.9%	0.094	0.92	0.83	1.02		--	--	--	--	--	--	27.9%	27.4%	0.3516	1.08	0.92	1.28	--	--
CR2 exon10	G	rs17615	207646462	A	G	27.2%	30.5%	0.011	0.87	0.78	0.97	0.35	36.7%	38.1%	0.32	0.94	0.82	1.07	21.2%	21.1%	0.4478	1.07	0.90	1.28	0.1578	0.95
CR2 intron10	I	rs148404045	207646661	A	T	--	--	--	--	--	--		2.4%	2.6%	0.84	0.96	0.63	1.45	--	--	--	--	--	--	--	--
CR2 exon11	G	rs4308977	207646898	C	T	27.5%	30.4%	0.021	0.88	0.79	0.98	0.50	48.4%	49.4%	0.58	0.97	0.85	1.10	22.6%	22.4%	0.4714	1.07	0.90	1.27	0.289	0.96
CR2 exon11	G	rs17616	207646923	A	G	27.0%	30.3%	0.011	0.87	0.78	0.97	0.36	36.5%	37.6%	0.38	0.94	0.83	1.08	20.5%	20.0%	0.2827	1.11	0.92	1.33	0.1856	0.95
CR2 intron12	I	rs9429775	207647339	G	A	--	--	--	--	--	--		22.7%	23.0%	0.93	1.01	0.86	1.18	1.9%	2.0%	0.5343	0.84	0.49	1.45	--	--
CR2 intron13	I	rs3991865	207647993	A	C	--	--	--	--	--	--		21.6%	22.0%	0.99	1.00	0.85	1.17	1.9%	2.0%	0.5343	0.84	0.49	1.45	--	--
CR2 exon14	G	rs61735651	207648173	C	T	6.9%	9.6%	9.1E-04	0.73	0.61	0.88	ND	10.4%	11.2%	0.25	0.89	0.72	1.09	4.6%	5.8%	0.1887	0.80	0.57	1.12	0.0004515	0.80
CR2 exon14	G	rs1143665	207648494	C	T	0.1%	0.1%	0.58	0.62	0.11	3.38		6.8%	6.4%	0.40	1.12	0.87	1.44	0.8%	0.4%	0.1617	2.04	0.75	5.53	--	--
CR2 intron14	I	1-207648720	207648720	A	ATTGT	--	--	--	--	--	--		3.9%	3.3%	0.42	1.15	0.82	1.61	--	--	--	--	--	--	--	--
CR2 intron14	I	rs9429776	207649224	A	G	--	--	--	--	--	--		18.2%	18.2%	0.76	1.03	0.87	1.22	--	--	--	--	--	--	--	--
CR2 intron14	I	rs143926411	207649434	C	G	--	--	--	--	--	--		1.7%	1.4%	0.47	1.21	0.72	2.02	--	--	--	--	--	--	--	--
CR2 intron15	I	rs9429777	207650191	G	A	--	--	--	--	--	--		11.3%	11.5%	0.93	0.99	0.81	1.22	--	--	--	--	--	--	--	--
CR2 intron15	I	rs9429938	207650464	C	A	--	--	--	--	--	--		18.2%	18.0%	0.66	1.04	0.88	1.23	--	--	--	--	--	--	--	--
CR2 intron15	I	rs150797291	207650498	G	T	--	--	--	--	--	--		3.4%	3.7%	0.63	0.92	0.65	1.30	--	--	--	--	--	--	--	--
CR2 intron15	G	rs7549152	207650938	T	G	6.9%	9.8%	5.7E-04	0.73	0.61	0.87	ND	10.8%	11.4%	0.36	0.91	0.74	1.11	4.6%	5.6%	0.2938	0.83	0.59	1.17	0.0009705	0.81
CR2 intron16	I	rs9429778	207651835	A	C	27.2%	30.4%	0.014	0.87	0.78	0.97	0.37	--	--	--	--	--	--	--	--	--	--	--	--	--	--
CR2 intron16	I	rs4844596	207651935	A	G	44.6%	46.5%	0.15	0.93	0.84	1.03		--	--	--	--	--	--	--	--	--	--	--	--	--	--
CR2 intron16	G	rs17045328	207652176	G	A	--	--	--	--	--	--		2.6%	2.1%	0.18	1.34	0.87	2.04	1.2%	1.3%	0.873	1.06	0.55	2.04	--	--
CR2 intron17	I	rs147029386	207652928	C	A	--	--	--	--	--	--		3.1%	3.7%	0.36	0.84	0.59	1.21	--	--	--	--	--	--	--	--
CR2 intron17	I	rs138313826	207652934	T	C	--	--	--	--	--	--		3.1%	3.7%	0.37	0.85	0.59	1.22	--	--	--	--	--	--	--	--
CR2 intron17	I	rs1475445	207652987	G	T	--	--	--	--	--	--		10.9%	11.1%	0.94	0.99	0.80	1.22	--	--	--	--	--	--	--	--
CR2 intron17	I	rs4317805	207652992	A	G	--	--	--	--	--	--		1.0%	1.1%	0.57	0.83	0.43	1.59	--	--	--	--	--	--	--	--
CR2 intron17	G	rs2182909	207653106	C	A	26.5%	29.7%	0.017	0.88	0.79	0.98	0.38	20.2%	22.0%	0.080	0.87	0.74	1.02	19.4%	19.5%	0.5161	1.06	0.88	1.28	0.1083	0.94
CR2 intron17	G	rs12032512	207653178	G	C	43.9%	46.8%	0.084	0.92	0.83	1.01		23.6%	25.1%	0.069	0.87	0.75	1.01	42.2%	39.8%	0.13	1.12	0.97	1.30	0.3538	0.97
CR2 exon18	G	rs17258982	207653364	G	A	7.8%	7.7%	1.00	1.00	0.83	1.20		9.2%	9.7%	0.43	0.92	0.74	1.14	4.5%	5.1%	0.7395	0.95	0.68	1.31	0.5487	0.96
CR2 exon18	I	rs6540433	207653395	C	A	--	--	--	--	--	--		1.8%	2.1%	0.26	0.76	0.47	1.23	--	--	--	--	--	--	--	--
CR2 intron18	I	rs17258996	207653444	A	T	19.8%	20.3%	0.63	0.97	0.86	1.10		6.2%	7.0%	0.20	0.84	0.64	1.10	14.5%	13.1%	0.06819	1.22	0.99	1.50	0.4921	1.03
CR2 intron18	I	rs7548495	207653701	A	T	6.7%	9.4%	7.5E-04	0.73	0.60	0.88	ND	--	--	--	--	--	--	3.8%	5.3%	0.1135	0.75	0.52	1.07	--	--
CR2 intron18	I	1-207653792	207653792	TG	T	19.7%	20.4%	0.50	0.96	0.85	1.08		20.3%	20.4%	0.96	1.00	0.85	1.17	15.4%	14.5%	0.2258	1.13	0.93	1.39	0.5843	1.02
CR2 intron18	I	1-207653793	207653793	GT	G	19.8%	20.6%	0.44	0.95	0.84	1.08		19.8%	20.2%	0.81	0.98	0.83	1.16	15.4%	14.5%	0.2258	1.13	0.93	1.39	0.6936	1.02
CR2 intron18	G	rs12117916	207653814	C	T	18.8%	19.9%	0.29	0.93	0.82	1.06		20.6%	20.2%	0.77	1.03	0.87	1.21	15.2%	14.7%	0.3766	1.10	0.89	1.35	0.7271	1.01
CR2 intron18	I	1-207654265	207654265	C	CA	18.7%	19.3%	0.61	0.97	0.85	1.10		--	--	--	--	--	--	13.7%	13.1%	0.3099	1.12	0.90	1.39	--	--
CR2 intron18	I	rs12059417	207654301	T	A	6.6%	9.4%	5.7E-04	0.72	0.60	0.87	ND	--	--	--	--	--	--	3.8%	5.2%	0.1485	0.77	0.53	1.10	--	--
CR2 intron18	I	rs17186771	207655193	G	C	21.4%	22.1%	0.50	0.96	0.85	1.08		6.1%	7.2%	0.10	0.80	0.61	1.05	13.9%	13.1%	0.1808	1.16	0.93	1.45	0.8741	1.01
CR2 intron18	I	rs11117884	207655472	G	A	42.6%	41.1%	0.17	1.07	0.97	1.18		--	--	--	--	--	--	--	--	--	--	--	--	--	--
CR2 intron18	I	rs1041250	207655815	A	G	21.6%	22.1%	0.66	0.97	0.87	1.10		6.2%	7.1%	0.19	0.84	0.64	1.09	15.0%	14.2%	0.1489	1.16	0.95	1.43	0.6242	1.02
CR2 intron18	G	rs6690215	207656050	C	T	42.1%	43.6%	0.19	0.94	0.85	1.03		57.1%	58.7%	0.38	0.94	0.83	1.08	33.6%	36.8%	0.2728	0.92	0.79	1.07	0.07436	0.95
CR2 intron18	G	rs7543913	207656502	T	C	21.8%	22.2%	0.74	0.98	0.87	1.10		7.0%	7.4%	0.55	0.93	0.72	1.19	15.1%	14.6%	0.2186	1.14	0.93	1.40	0.5023	1.03
CR2 intron18	G	rs6665881	207656605	C	G	42.6%	41.0%	0.16	1.07	0.97	1.18		37.0%	34.7%	0.24	1.08	0.95	1.24	55.0%	52.5%	0.729	1.03	0.89	1.19	0.2129	1.04
CR2 intron18	I	rs61821133	207657173	A	C	6.1%	8.0%	0.017	0.79	0.65	0.96	0.34	--	--	--	--	--	--	4.0%	5.0%	0.264	0.81	0.57	1.17	--	--
CR2 intron18	G	rs17045468	207657375	T	C	0.9%	0.9%	0.44	0.81	0.48	1.38		1.9%	1.7%	0.62	1.13	0.70	1.80	0.7%	0.8%	0.9939	1.00	0.42	2.35	0.14	1.10
CR2 intron18	G	rs12021671	207658145	A	G	35.3%	36.6%	0.24	0.94	0.85	1.04		56.1%	57.8%	0.43	0.95	0.83	1.08	29.7%	32.1%	0.3372	0.93	0.79	1.08	0.1254	0.95
CR2 intron18	I	rs71635141	207658395	T	C	7.1%	7.4%	0.99	1.00	0.83	1.21		1.6%	1.4%	0.76	1.09	0.64	1.86	2.9%	2.3%	0.06553	1.54	0.97	2.45	--	--
CR2 intron18	I	rs1977465	207658507	A	G	35.0%	36.5%	0.17	0.93	0.84	1.03		55.6%	57.3%	0.47	0.95	0.83	1.09	28.5%	30.7%	0.3939	0.93	0.79	1.10	0.09951	0.95
CR2 intron19	I	rs9429939	207659617	G	A	--	--	--	--	--	--		1.8%	2.1%	0.79	0.94	0.58	1.51	--	--	--	--	--	--	--	--
CR2 intron19	I	1-207659913	207659913	CTG	C	18.4%	19.4%	0.24	0.93	0.82	1.05		--	--	--	--	--	--	14.9%	16.1%	0.5109	0.94	0.77	1.14	--	--

CR2 intron19	G	rs2182911	207660071	C	T	18.5%	19.5%	0.23	0.93	0.82	1.05		12.0%	13.3%	0.38	0.92	0.76	1.11	14.5%	15.9%	0.4067	0.92	0.75	1.12	0.0723	0.92
CR2 intron19	I	rs2182912	207660344	T	A	18.5%	19.5%	0.25	0.93	0.82	1.05		4.9%	5.8%	0.17	0.82	0.61	1.09	14.4%	15.8%	0.4589	0.93	0.76	1.13	0.07052	0.92
CR2 intron19	G	rs2182913	207660378	A	G	29.6%	32.6%	0.021	0.88	0.80	0.98	0.24	50.8%	51.5%	0.61	0.97	0.85	1.10	24.4%	27.2%	0.1898	0.90	0.76	1.06	0.009686	0.91
CR2 intron19	G	rs7519408	207661289	G	C	12.0%	12.1%	0.76	0.98	0.84	1.14		11.2%	12.7%	0.44	0.92	0.76	1.13	11.1%	12.1%	0.3976	0.91	0.72	1.14	0.2659	0.94
CR2 intron19	I	rs115749816	207661321	A	G	--	--	--	--	--	--		4.2%	5.0%	0.72	0.94	0.69	1.30	--	--	--	--	--	--	--	--
CR2 intron19	I	rs61821134	207661446	G	A	6.3%	8.2%	0.018	0.79	0.65	0.96	0.18	--	--	--	--	--	--	3.4%	4.4%	0.239	0.79	0.54	1.17	--	--
CR2 intron19	I	rs76484450	207661485	G	A	5.2%	5.4%	0.84	0.98	0.79	1.21		6.6%	6.4%	0.87	1.02	0.78	1.33	3.7%	4.2%	0.8324	0.96	0.66	1.39	--	--
CR2 intron19	I	rs79578547	207661637	G	A	--	--	--	--	--	--		4.1%	3.5%	0.47	1.13	0.81	1.58	--	--	--	--	--	--	--	--
CR2 intron19	G	rs4618970	207661640	C	T	11.8%	12.3%	0.42	0.94	0.81	1.09		11.1%	12.6%	0.39	0.92	0.75	1.12	10.8%	12.0%	0.3032	0.89	0.71	1.11	0.1124	0.92
CR2 intron19	I	rs61821135	207661679	C	T	6.3%	8.2%	0.018	0.79	0.65	0.96	0.18	--	--	--	--	--	--	3.4%	4.4%	0.239	0.79	0.54	1.17	--	--
CR2 intron19	I	rs147742969	207661861	T	C	4.3%	4.7%	0.59	0.94	0.74	1.19		6.6%	6.4%	0.87	1.02	0.78	1.33	3.7%	4.2%	0.8324	0.96	0.66	1.39	--	--
CR2 intron19	I	rs12068225	207662306	A	G	--	--	--	--	--	--		5.7%	5.7%	0.82	1.04	0.78	1.38	--	--	--	--	--	--	--	--
CR2 3'UTR	I	rs9429940	207662912	T	C	11.8%	12.3%	0.41	0.94	0.81	1.09		11.1%	12.6%	0.41	0.92	0.75	1.12	10.8%	11.7%	0.4035	0.91	0.73	1.14	0.1397	0.93
CR1 5' upstream	G	rs17045761	207663356	A	G	6.5%	5.1%	0.044	1.23	1.01	1.51	0.08	4.9%	5.3%	0.47	0.90	0.67	1.20	6.1%	5.6%	0.4241	1.13	0.83	1.54	0.9122	1.01
CR1 5' upstream	I	rs4525038	207663937	T	C	11.8%	12.4%	0.40	0.94	0.81	1.09		--	--	--	--	--	--	12.2%	12.7%	0.56	0.94	0.75	1.17	--	--
CR1 5' upstream	I	rs6663887	207664423	A	G	23.0%	25.4%	0.043	0.89	0.80	1.00	0.39	--	--	--	--	--	--	20.6%	22.9%	0.181	0.89	0.74	1.06	--	--
CR1 5' upstream	I	rs6700109	207664607	C	T	16.1%	17.0%	0.31	0.93	0.82	1.06		--	--	--	--	--	--	15.2%	16.6%	0.4029	0.92	0.75	1.12	--	--
CR1 5' upstream	I	rs9429779	207664721	G	A	29.6%	32.6%	0.018	0.88	0.79	0.98	0.21	50.6%	51.6%	0.48	0.95	0.84	1.09	24.1%	27.0%	0.174	0.89	0.76	1.05	0.006757	0.91
CR1 5' upstream	I	rs61821136	207665084	G	C	6.3%	8.2%	0.018	0.79	0.65	0.96	0.18	--	--	--	--	--	--	3.4%	4.4%	0.2361	0.79	0.54	1.16	--	--
CR1 5' upstream	I	rs115883226	207665226	G	A	--	--	--	--	--	--		4.2%	5.0%	0.71	0.94	0.69	1.29	--	--	--	--	--	--	--	--
CR1 5' upstream	I	rs10863336	207665920	T	C	32.6%	30.9%	0.24	1.07	0.96	1.18		--	--	--	--	--	--	--	--	--	--	--	--	--	--
CR1 5' upstream	G	rs4618971	207666061	C	T	23.2%	24.2%	0.29	0.94	0.84	1.05		26.3%	27.7%	0.51	0.95	0.82	1.10	19.5%	21.3%	0.4259	0.93	0.78	1.11	0.118	0.94
CR1 5' upstream	I	rs1410408	207666308	A	G	6.9%	5.7%	0.15	1.16	0.95	1.41		6.6%	7.7%	0.24	0.86	0.67	1.11	6.0%	5.6%	0.452	1.12	0.83	1.53	--	--
CR1 5' upstream	I	rs11807805	207666597	G	C	4.2%	4.4%	0.80	0.97	0.76	1.23		--	--	--	--	--	--	3.8%	4.1%	0.9815	1.00	0.69	1.46	--	--
CR1 5' upstream	I	rs9429780	207667190	G	C	22.7%	24.1%	0.19	0.93	0.83	1.04		--	--	--	--	--	--	19.4%	20.7%	0.6445	0.96	0.80	1.15	--	--
CR1 5' upstream	I	rs9429781	207667222	G	T	22.7%	24.1%	0.19	0.93	0.83	1.04		--	--	--	--	--	--	18.8%	20.4%	0.5276	0.94	0.78	1.13	--	--
CR1 5' upstream	I	rs9429941	207667303	T	A	22.7%	24.1%	0.19	0.93	0.83	1.04		--	--	--	--	--	--	19.4%	20.6%	0.6804	0.96	0.80	1.16	--	--
CR1 5' upstream	I	rs115257007	207667495	A	C	3.3%	3.5%	0.76	0.96	0.73	1.25		--	--	--	--	--	--	3.8%	4.0%	0.9283	1.02	0.70	1.48	--	--
CR1 5' upstream	I	rs12078329	207667497	T	C	4.3%	4.5%	0.83	0.98	0.77	1.23		--	--	--	--	--	--	5.2%	4.8%	0.4643	1.13	0.81	1.59	--	--
CR1 5' upstream	I	1-207667501	207667501	G	GA	3.6%	3.7%	0.85	0.98	0.75	1.27		--	--	--	--	--	--	--	--	--	--	--	--	--	--
CR1 5' upstream	I	rs76124527	207667549	G	A	4.3%	4.5%	0.83	0.98	0.77	1.23		13.7%	13.7%	0.87	1.02	0.84	1.23	4.7%	4.5%	0.5187	1.12	0.79	1.58	--	--
CR1 5' upstream	I	rs11117911	207667985	A	G	4.4%	4.5%	0.90	0.98	0.78	1.25		--	--	--	--	--	--	5.2%	4.8%	0.4643	1.13	0.81	1.59	--	--
CR1 5' upstream	I	rs11117913	207668235	T	C	4.3%	4.5%	0.83	0.98	0.77	1.23		--	--	--	--	--	--	5.2%	4.8%	0.4571	1.14	0.81	1.59	--	--
CR1 5' upstream	I	rs80141998	207668315	A	G	4.2%	4.4%	0.72	0.96	0.75	1.22		6.4%	5.9%	0.62	1.07	0.82	1.40	3.8%	4.0%	0.9217	1.02	0.70	1.48	--	--
CR1 5' upstream	I	rs7525160	207668414	C	G	33.3%	31.5%	0.18	1.07	0.97	1.19		--	--	--	--	--	--	--	--	--	--	--	--	--	--
CR1 5' upstream	I	rs7525170	207668447	G	A	18.3%	19.5%	0.17	0.92	0.81	1.04		--	--	--	--	--	--	14.1%	15.6%	0.428	0.92	0.75	1.13	--	--
CR1 5' upstream	I	1-207668549	207668549	T	TTTTC	4.2%	4.4%	0.72	0.96	0.75	1.22		6.4%	6.0%	0.71	1.05	0.80	1.38	3.8%	4.0%	0.9217	1.02	0.70	1.48	--	--
CR1 5' upstream	I	rs9429942	207668630	C	T	--	--	--	--	--	--		50.5%	51.2%	0.57	0.96	0.85	1.10	23.8%	26.7%	0.1861	0.89	0.75	1.06	--	--
CR1 5' upstream	I	rs55819427	207669111	T	C	6.2%	8.2%	0.016	0.79	0.65	0.96	0.19	--	--	--	--	--	--	4.6%	5.6%	0.3017	0.84	0.60	1.17	--	--
CR1 5' upstream	I	rs61522287	207669248	A	G	4.2%	4.4%	0.72	0.96	0.75	1.22		6.4%	6.0%	0.68	1.06	0.81	1.38	3.8%	4.0%	0.9217	1.02	0.70	1.48	--	--
CR1 5' upstream	I	rs61821138	207669343	A	G	11.0%	12.9%	0.070	0.87	0.75	1.01		--	--	--	--	--	--	9.8%	10.8%	0.5405	0.93	0.73	1.18	--	--
CR1 intron1	I	rs9429943	207669924	C	G	22.7%	25.1%	0.051	0.89	0.80	1.00		49.4%	50.5%	0.48	0.96	0.84	1.09	20.3%	22.3%	0.2748	0.91	0.76	1.08	0.02154	0.92
CR1 intron1	I	rs115050353	207670281	C	A	4.2%	4.4%	0.77	0.96	0.76	1.23		6.4%	6.0%	0.71	1.05	0.81	1.38	3.8%	4.0%	0.9061	1.02	0.70	1.49	--	--
CR1 intron1	I	rs111449973	207670307	T	A	22.7%	25.1%	0.052	0.89	0.80	1.00		49.3%	50.5%	0.45	0.95	0.84	1.08	20.3%	22.3%	0.2748	0.91	0.76	1.08	0.02027	0.92
CR1 intron1	G	rs112822892	207670438	G	A	29.8%	30.2%	0.92	0.99	0.90	1.11		8.9%	9.4%	0.52	0.93	0.74	1.16	18.5%	17.7%	0.08811	1.18	0.98	1.42	0.4029	1.03
CR1 intron1	G	rs113977956	207670744	C	T	11.0%	12.9%	0.071	0.87	0.75	1.01		38.4%	37.9%	0.93	0.99	0.87	1.13	9.9%	11.0%	0.511	0.92	0.73	1.17	0.1605	0.94
CR1 intron1	G	rs113151130	207670928	G	A	10.6%	12.6%	0.056	0.86	0.74	1.00		38.1%	37.6%	1.00	1.00	0.88	1.14	9.6%	10.5%	0.6929	0.95	0.75	1.21	0.1933	0.94
CR1 intron1	G	rs112585529	207671269	C	T	18.0%	19.4%	0.16	0.91	0.81	1.04		2.5%	2.5%	0.52	0.87	0.58	1.32	13.1%	14.9%	0.3049	0.90	0.73	1.10	0.05632	0.91
CR1 intron1	G	rs113901708	207671476	C	T	18.1%	19.1%	0.24	0.93	0.82	1.05		7.8%	8.4%	0.38	0.90	0.71	1.14	13.7%	15.1%	0.4075	0.92	0.75	1.13	0.08226	0.92
CR1 intron1	I	rs115808230	207671621	A	G	6.2%	8.1%	0.016	0.79	0.65	0.96	0.19	--	--	--	--	--	--	3.4%	4.4%	0.2302	0.79	0.54	1.16	--	--
CR1 intron1	G	rs112981697	207671849	A	C	11.1%	12.8%	0.10	0.88	0.76	1.03		38.4%	38.0%	0.92	0.99	0.87	1.13	9.9%	10.9%	0.5472	0.93	0.74	1.18	--	--
CR1 intron1	I	rs114715994	207672533	T	C	--	--	--	--	--	--		3.1%	3.8%	0.13	0.75	0.52	1.09	--	--	--	--	--	--	--	--
CR1 intron1	I	rs113379492	207672612	G	A	22.8%	25.1%	0.058	0.90	0.80	1.00		49.3%	50.6%	0.45	0.95	0.84	1.08	20.2%	22.4%	0.2099	0.89	0.75	1.07	0.01866	0.91
CR1 intron1	I	rs61822962	207672719	T	G	11.1%	12.8%	0.091	0.88	0.76	1.02		38.2%	38.0%	0.80	0.98	0.86	1.12	9.8%	10.9%	0.5324	0.93	0.73	1.17	--	--
CR1 intron1	I	rs59179545	207672988	C	G	11.0%	12.8%	0.084	0.88	0.75	1.02		38.2%	38.0%	0.80	0.98	0.86	1.12	9.8%	10.9%	0.5324	0.93	0.73	1.17	--	--
CR1 intron1	I	rs112381793	207673084	A	T	22.8%	25.1%	0.055	0.89	0.80	1.00		49.3%	50.6%	0.42	0.95	0.83	1.08	20.1%	22.4%	0.1996	0.89	0.75	1.06	0.01691	0.91
CR1 intron1	I	rs61822963	207673414	A	G	6.1%	8.1%	0.014	0.78	0.65	0.95	0.20	--	--	--	--	--	--	3.7%	4.8%	0.229	0.80	0.55	1.15	--	--
CR1 intron1	I	rs112049645	207673631	T	C	23.0%	25.2%	0.069	0.90	0.80	1.01		49.2%	50.5%	0.42	0.95	0.83	1.08	20.1%	22.4%	0.1996	0.89	0.75	1.06	0.02017	0.92
CR1 intron1	I	rs113012055	207673945	A	G	18.0%	19.3%	0.18	0.92	0.81	1.04		2.2%	2.4%	0.30	0.80	0.52	1.23	13.5%	15.4%	0.2532	0.89	0.72	1.09	0.04975	0.91
CR1 intron1	G	rs113703567	207673980	A	G	11.1%	12.9%	0.093	0.88	0.75	1.02		37.7%	38.0%	0.60	0.97	0.85	1.10	10.0%	11.0%	0.5536	0.93	0.74	1.18	0.1071	0.93
CR1 intron1	I	rs114467562	207674503	T	C	--	--	--	--	--	--		1.1%	1.4%	0.43	0.78	0.43	1.43	--	--	--	--	--	--	--	--
CR1 intron1	G	rs112741151	207675655	A	G	30.2%	30.5%	0.94	1.00	0.90	1.12		24.3%	24.2%	0.68	1.03	0.89	1.20	20.3%	19.1%	0.05588	1.20	1.00	1.44	0.1579	1.05
CR1 intron1	I	rs61822964	207675712	G	A	6.1%	8.1%	0.017	0.79	0.65	0.96	0.22	--	--	--	--	--	--	--	--	--	--	--	--	--	--
CR1 intron1	I	rs111445330	207675853	G	T	11.0%	12.7%	0.088	0.88	0.75	1.02		37.8%	37.8%	0.74	0.98	0.86	1.12	9.6%	10.8%	0.4623	0.91	0.72	1.16	--	--
CR1 intron1	I	rs61822965	207676203	G	A	5.5%	7.1%	0.030	0.80	0.65	0.98	0.23	--	--	--	--	--	--	--	--	--	--	--	--	--	--

Abbreviations: Freq, allele frequency; OR, odds ratio; CI, confidence interval; P_c, P value after conditioning on rs1876453; ND, not determined; I, imputed; G, genotyped; CTRL, control; EA, European American; AA, African American; AS, Asian; HS, Hispanic; Meta, Meta-analysis

Position is based on GRCh37/hg19.
